# Prioritizing Conservation of Trailing‐Edge Populations for Future Climate‐Resilient Forests

**DOI:** 10.1111/gcb.70971

**Published:** 2026-06-25

**Authors:** Nicholas Boyce, Andreas Hamann, Genevieve Dorrell, Scott E. Nielsen

**Affiliations:** ^1^ Department of Renewable Resources, Faculty of Agricultural, Life, and Environmental Sciences University of Alberta Edmonton Alberta Canada

**Keywords:** adaptive management, assisted migration, climate change, conservation planning, forest management, genetic conservation

## Abstract

Trailing edge tree populations, located at the warm or dry margins of species' ranges, often harbor unique genetic adaptations to marginal environments. These populations face heightened risks from climate change, potentially threatening the persistence of valuable adaptive traits. Here we identify trailing‐edge populations for the 100 most common North American tree species using spatially explicit climate and forest inventory data, and prioritize conservation actions based on projected forest cover loss, climate velocity, and species richness. Assisted migration initiatives for these populations could safeguard the long‐term persistence of their adaptive traits, ensuring that valuable genotypes are maintained within future forest ecosystems. Trailing‐edge populations were geographically concentrated in the Appalachian region, the Midwest, and southern boreal forests. Areas bordering the central plains faced the highest projected forest cover loss and climate velocity, while the Great Lakes basin and eastern Canada emerged as promising recipient regions. Our findings support targeted conservation and assisted migration strategies to maintain genetic diversity and enhance forest resilience under future climates, facilitated by an online tool for climate‐informed seed collection and planning (https://tinyurl.com/past‐nam).

## Graphical abstract

Climate change threatens warm‐ and dry‐edge tree populations that may harbor adaptive genetic variation important for future forest resilience. We identified trailing‐edge populations for frequent and widespread North American tree species using climate and forest inventory data, then prioritized conservation actions based on projected forest habitat loss, climate velocity, and conservation value. Results are linked to https://tinyurl.com/past‐nam, a decision‐support tool for identifying suitable protected areas and guiding seed collection, conservation, and assisted migration planning.

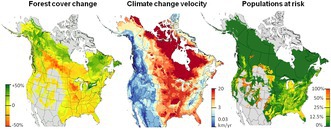

## 1 Introduction

Trailing edge tree populations, occurring at the warmest or driest parts of a species' range, are focal points for conservation under climate change. Under shifting climates, these populations face range contractions or local extirpation, either because populations are outcompeted by species adapted to warmer environments (e.g., Gilman et al. 2010) or due to direct climate impacts when climates exceed physiological limits (e.g., Hammond et al. 2022). Trailing‐edge populations are often genetically distinct, having evolved under frequent exposure to climatic extremes such as heat, drought, or otherwise marginal conditions (Hampe and Petit 2005; Lesica and Allendorf 1995; Pelletier et al. 2023; Perrier et al. 2026). Under climate warming, such locally adapted populations with unique heat and drought tolerance traits become potentially valuable, as larger portions of a species' current range begin to resemble the historical climate of its warm/dry edge. They may serve as important reservoirs of pre‐adapted genotypes that can enhance resilience in other parts of the species' range, when used in assisted gene flow or assisted migration management strategies (Aitken and Whitlock 2013).

In some cases, trailing‐edge populations exhibit lower genetic diversity within individual populations due to small population sizes and strong selective pressures. However, genetic differentiation between these populations is typically high (Hampe and Petit 2005; Perrier et al. 2026). This can be especially pronounced in isolated populations where local adaptation can occur rapidly, leading to the emergence of distinct ecotypes. These isolated populations are most likely to harbor unique combinations of adaptive traits that are potentially valuable for enhancing climate resilience elsewhere (Macdonald et al. 2017; Perrier et al. 2026). In other cases, trailing‐edge populations persist along elevation gradients where connectivity is maintained over time (Hampe and Petit 2005). Here, environmental heterogeneity and gene flow can sustain high within‐population genetic diversity, providing strong evolutionary potential for future adaptation. Both types of trailing‐edge populations, whether isolated and rapidly evolving or connected and genetically diverse, may harbor value for conservation and adaptive management. For example, a recent study on jack pine ( *Pinus banksiana* ) found that trailing‐edge populations displayed lower and more variable serotiny than core populations, an adaptive trait that may enhance population resilience in regions with infrequent fire regimes (Pelletier et al. 2023). Adaptive traits such as these, along with the observed genetic differentiation among trailing‐edge populations, suggest they may be valuable targets for genetic conservation even if the species as a whole is not currently threatened.

Alongside their evolutionary significance, trailing‐edge forests in North America face heightened threats due to the combined impacts of climate change, altered disturbance regimes, and human land use (e.g., Parks et al. 2019; Rodman et al. 2022). Recent findings already show evidence of present‐day climate warming pushing these already water‐limited ecosystems beyond their historical thresholds, leading to regeneration failure and increased tree mortality (e.g., Rodman et al. 2022; Worrall et al. 2013). In the eastern and central parts of North America, trailing‐edge tree populations often occupy low‐elevation sites with productive soils and warmer climates (Parks et al. 2019), the same landscapes that have largely been converted to agriculture or have been subject to urban development. This habitat loss and fragmentation from land‐use change further isolate and reduce the viability of warm‐adapted tree populations (Rhoades et al. 2024).

Furthermore, observed and projected climate change is expected to impact populations of many North American tree species. Climate envelope models predict that populations are already experiencing a lag between their historical climatic niches and current local climates, estimated at approximately 310 km in latitude or 140 m in elevation as of the 2020s (Gray and Hamann 2013). While species as a whole may persist, populations at the trailing edge are among the first to encounter conditions that exceed their physiological limits. For example, trembling aspen ( *Populus tremuloides* ) has experienced widespread branch dieback and above‐ground mortality in trailing‐edge habitats where climatic suitability has declined (Worrall et al. 2013). Although climatic maladaptation may not immediately result in local extirpation and range contraction for all species, populations will face increased exposure to climate extremes, which can impact their resilience and competitive capacity.

Given these risk factors, it is unlikely that natural mechanisms such as gene flow, seed dispersal, and evolutionary processes will allow tree populations to cope with the rate of observed and projected climate change (Aitken et al. 2008). Human‐assisted migration and assisted gene flow have therefore emerged as important conservation and management strategies to mitigate maladaptation and preserve genetic diversity in forest trees (Aitken and Whitlock 2013; Aitken et al. 2008; Sáenz‐Romero et al. 2016; Williams and Dumroese 2013; Zou et al. 2024). Several authors have emphasized the importance of incorporating trailing‐edge populations into conservation planning, due to their unique adaptive traits and potential value for future forest resilience (Hampe and Petit 2005; Aitken and Whitlock 2013; Sáenz‐Romero et al. 2016).

Forest genetic conservation and climate adaptation programs are already underway at various jurisdictional levels, providing an applied context for conserving trailing‐edge populations. In Canada, the National Tree Seed Centre and the National Forest Genetic Resource Centre coordinate in situ and ex situ conservation of forest genetic resources, including climatically marginal populations (Loo et al. 2007; Natural Resources Canada 2023). Provincial programs in British Columbia and Alberta have also implemented climate‐based seed transfer guidelines, seed orchards, and conservation collections (Chourmouzis et al. 2009; Government of Alberta 2018). In the United States, government agencies and non‐profit organizations similarly maintain seed collections and conservation areas intended to preserve adaptive variation (Beardmore et al. 2015; USDA 2017). Taken together, these institutional frameworks show that forest genetic conservation capacity already exists across North America. Our study is intended to support these programs by providing a spatially explicit assessment of where trailing‐edge populations occur, where climate‐related risks may be greatest, and where conservation or assisted migration actions could be considered.

Here, we address this need by developing a systematic and spatially explicit analysis that identifies trailing‐edge populations for frequent and widespread tree species across North America. Rare or highly range‐restricted species are not excluded from the accompanying conservation tool, but they were not included in the trailing‐edge population analysis because they often lack clearly distinguishable trailing‐edge populations. The results presented here are intended to support government agencies and conservation organizations by informing priorities for in situ and ex situ conservation, guiding seed collection and deployment under changing climates, and supporting decisions on where to invest resources for conserving adaptive genetic variation in forest trees.

## 2 Methods

### 2.1 Ecosystem Delineations

To make our findings accessible in any jurisdiction, our analysis was based on the most widely used ecosystem delineations in specific jurisdictions to identify source populations and potential in situ climate change refugia. In British Columbia, we used Variant units from the Biogeoclimatic Ecosystem Classification (BEC) system, which provides a detailed and climatically grounded ecological framework (British Columbia Ministry of Forests 2024). In Alberta, the finest seed zone units of the Natural Regions and Subregions classification (Natural Regions Committee 2006) were used. For the rest of Canada, we used the Terrestrial Ecozones, Ecoregions, and Ecodistricts dataset developed by Agriculture and Agri‐Food Canada, selecting Ecodistricts (Agriculture and Agri‐Food Canada 2013). For the United States, we used the U.S. Environmental Protection Agency's Level IV Ecoregions, the national standard for fine‐scale ecological delineation (U.S. EPA 2013). We used Level III EPA ecoregions in Alaska, where Level IV delineations are unavailable. Ecosystems with an internal mean annual temperature range exceeding 4°C were subdivided into 2°C bands, a resolution chosen to reflect potential genetic differentiation and potential changes in species composition along elevation gradients. With the additional elevation bands, we used 2270 ecosystem units that are reasonably homogeneous in climate conditions and tree species composition.

### 2.2 Climate Data

To characterize past and future climatic conditions across ecosystems, we used 11 biologically relevant bioclimatic variables derived from monthly temperature and precipitation data using the ClimateNA software (Wang et al. 2016). The 11 bioclimatic variables selected for analysis are climatic factors known to influence plant distribution, productivity, and phenology: Mean Annual Temperature (MAT), Mean Warmest Month Temperature (MWMT), Mean Coldest Month Temperature (MCMT), and Temperature Difference (TD), calculated as MWMT–MCMT to represent continentality. We also included Extreme Minimum Temperature (EMT), defined as the coldest temperature expected over a 30‐year period, a potential driver of cold‐hardiness adaptations. Precipitation variables included Mean Annual Precipitation (MAP), Growing Season Precipitation from May to September (MSP), Precipitation as Snow (PAS), and a Climate Moisture Index (CMI), which integrates heat and moisture availability. Thermal indices, including Chilling Degree Days below 0°C (DD0) and the Number of Frost‐Free Days (NFFD), represent thresholds critical for plant growth and dormancy.

Historical climate data were obtained for the 1960s normal period (1951–1980), while medium‐term future projected climate data were based on the 2050s normal period (2041–2070). This period provides a realistic planning horizon for conservation actions initiated in the near term, whereas transfers aimed at late‐century climates may place populations in locations where frost risk remains too high for successful establishment. Future climate projections were based on an ensemble of eight CMIP6 Global Climate Models (ACCESS‐ESM1‐5, CNRM‐ESM2‐1, EC‐Earth3, GFDL‐ESM4, GISS‐E2‐1‐G, MIROC6, MPI‐ESM1‐2‐HR, MRI‐ESM2‐0) selected by Mahony et al. (2022) based on various quality criteria and overall representation of median climate change projections. Climate surfaces of historical and future climate conditions were generated at a spatial resolution of 1 km^2^ and aggregated by ecosystem units. These average climate values for ecosystems formed the basis for subsequent analyses, including climate analog comparisons among ecosystems from past (source) and future (target) climate conditions.

### 2.3 Tree Species Data

Tree species composition was estimated using data from two major national forest monitoring programs: Canada's National Forest Inventory (NFI) (Beaudoin et al. 2014) and the equivalent US forest inventory, using the same methodological approach (Wilson et al. 2012). Because Wilson et al. (2012) did not include Alaska, we used plot data from the U.S. Forest Inventory and Analysis (FIA) program (Gray et al. 2012) for this region. For Alaska, species basal area was summed across all measured trees per plot and averaged across measurement years. For plot‐based (FIA) and spatial (NFI, U.S.) datasets, species‐level proportional basal area was aggregated within each ecosystem delineation, scaled to relative species compositions that sum to one. To ensure taxonomic consistency across datasets, we harmonized species names using Latin binomials and verified them against common names where unambiguous matches existed. Species codes from Little's Atlas of United States Trees (Little 1971) were appended to all species records to facilitate integration with U.S. forestry databases, including the Silvics of North America reference (Burns and Honkala 1990), which uses the same nomenclature system.

To describe ecosystem suitability for tree species independent of human land use, we estimated potential natural forest cover excluding agricultural and urban conversions. We used the MODIS land cover product from the North American Land Change Monitoring System (Commission for Environmental Cooperation 2005) with a deep neural network classifier to replace agricultural and urban pixels with the most probable natural class based on climate and topography, yielding a reconstruction of potential natural land cover (Appendix S1). Reconstructed forest cover estimates were then used to scale species frequencies from the forest inventories. For each ecosystem, relative abundances of all tree species were rescaled so that forested and naturally non‐forested components together summed to one, enabling comparison of potential climate habitat among ecosystems with differing present‐day forest extent.

### 2.4 Identifying Trailing‐Edge Populations at Risk

We used an ecosystem‐level nearest‐neighbour bioclimatic envelope matching approach. Future climate conditions (2050s, 2041–2070) were matched to the most similar historical climates (1960s, 1951–1980) using a standardized Euclidean distance matrix based on 11 bioclimatic variables. We identified the five most similar historical analogs for each ecosystem under the projected climate and inferred future species composition by averaging species frequencies equally across these analogs. Averaging across the five closest analogues, similar to k‐nearest‐neighbour (kNN) imputation, reduces the influence of outliers and improves robustness, particularly for smaller ecosystems or those delineated based on distinctive bedrock or soil conditions rather than climate alone. In such cases, species composition may reflect limited local inventory data or local edaphic effects rather than macro‐climatic suitability.

Populations currently present within an ecosystem were classified as trailing edge if their projected future frequency fell below a species‐specific frequency threshold, defined as the 15th percentile of average ecosystem abundance within the species' historical distribution. We selected the 15th percentile empirically because it consistently identified marginal populations representing approximately 0.1% of each species' total basal area across its range. To further restrict results to climatically marginal sites, we retained only populations occurring within the warmest or driest 10% of ecosystems occupied by each species, based on mean annual temperature or climatic moisture deficit. This selection process defined the set of trailing‐edge populations used for subsequent risk assessment and conservation prioritization analyses.

Finally, we applied a dual validation filter to ensure that trailing‐edge populations represent actual occurrences. Each trailing‐edge population was required to have confirmed forest inventory records within the ecosystem and to fall within or near its native range (buffered by 50 km) based on historical range maps from Little (1971). Only ecosystems meeting both criteria were retained as climatically vulnerable trailing‐edge populations suitable for targeted seed collection and conservation planning. Further methodological detail is provided in Appendix S2.

### 2.5 Evaluating Conservation Need and Opportunity

We carried out a risk and value assessment of trailing‐edge populations using three complementary criteria: (1) the severity of climate‐driven risk of local population extirpation in the short term, (2) the potential for natural dispersal or gene flow to maintain genetic diversity versus the need for human intervention, and (3) the overall conservation value of each ecosystem in terms of species richness and genetic diversity potentially at risk.

Projected forest loss between the 1960s baseline and the 2050s was derived from reconstructed potential forest cover using the ecosystem climate‐matching approach described above. Because suitable habitat for all 100 tree species is considered simultaneously, this metric can be interpreted as a proxy for fundamental climatic limits on tree growth and regeneration in general, representing all tree species' joint realized climate niches. Declines in forest cover, therefore, may indicate that future conditions could exceed species' physiological tolerances, providing a valuable indicator of climate‐driven risk of local population extirpation.

To assess the need for human intervention, climate velocity was calculated following Loarie et al. (2009) and implemented using the distance‐based analogue‐matching method of Hamann et al. (2015), which identifies the nearest future climate analogue for each location. High velocities suggest that natural dispersal or gene flow may be insufficient for tree species to remain within their climate envelope, implying a need for assisted migration, while low velocities indicate an opportunity for genes to persist through gene flow and short‐range dispersal along elevation gradients.

Conservation value was expressed as the number and proportion of species with trailing‐edge populations in each ecosystem, identifying areas with a high concentration of at‐risk populations.

We further evaluated potential sites for in situ conservation and assisted migration across North America. We quantified two metrics for each ecosystem: (1) demand, the number of trailing‐edge populations for which an ecosystem's projected 2050 climate was identified as a suitable analogue; and (2) capacity, the area of protected land with climate habitat suitable for forest trees by the 2050s. Protected area data were obtained from the World Database on Protected Areas (UNEP‐WCMC 2025) and limited to sites recognized by the International Union for Conservation of Nature (IUCN) for data quality assurance.

## 3 Results

### 3.1 Identifying Trailing‐Edge Populations at Risk

**FIGURE 1:**
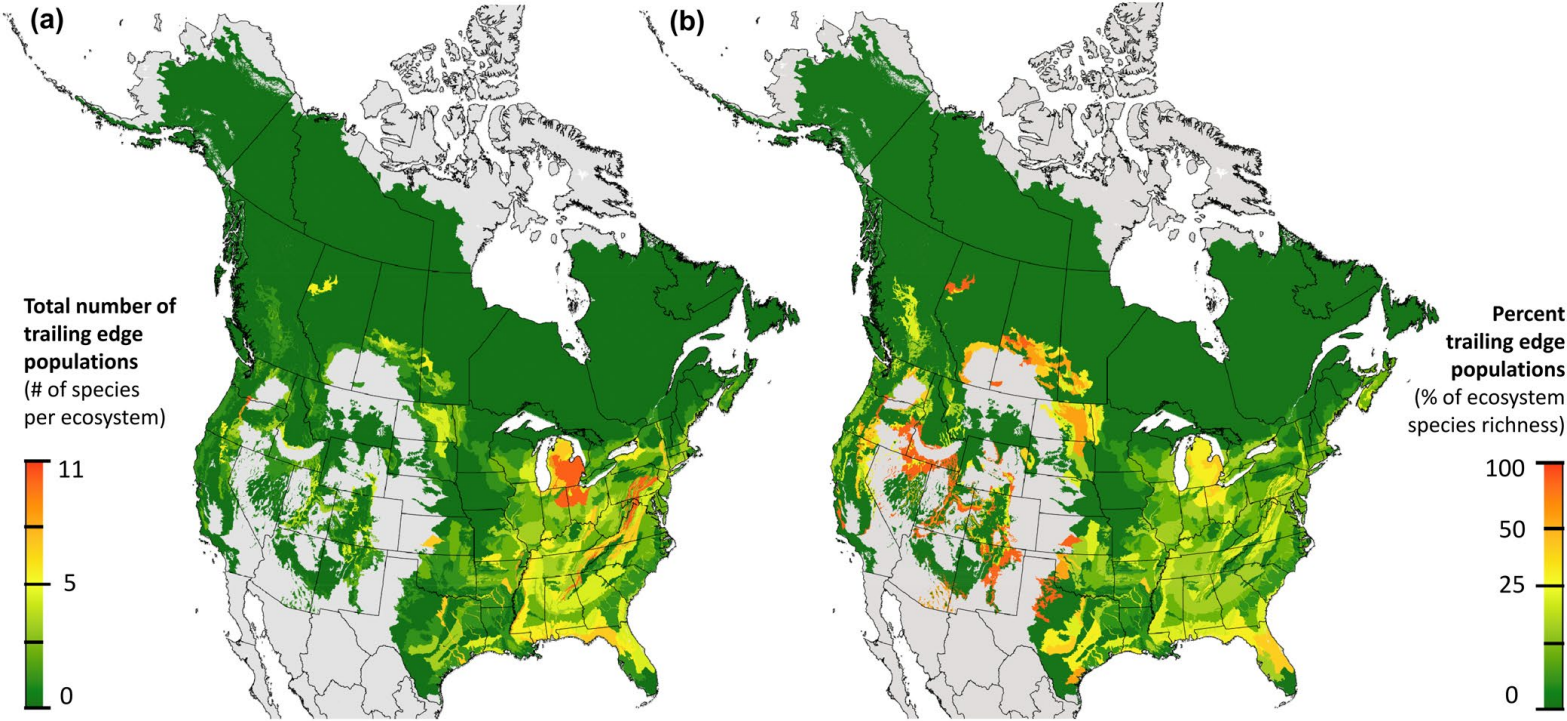
Trailing edge tree populations at warm or dry range margins are at heightened risk due to climate change. (a) The total count of at‐risk populations as the number of threatened species in each ecosystem. (b) Climate threat as the proportion of at‐risk species to all study species in the ecosystem.

We summarized the importance of potential losses across ecosystems based on the number and proportion of species with at‐risk populations. Figure 1 highlights ecosystems where trailing‐edge species populations are concentrated with projected loss of suitable climate habitat by the 2050s. In terms of the absolute number of trailing‐edge populations (Figure 1a), the Appalachian Mountains and the Great Lakes regions have the highest counts. These regions are species‐rich and represent the trailing edges of many northern tree species. Mapping the relative proportion of trailing‐edge populations (Figure 1b) highlights regions such as the southern boreal‐temperate transition zones, lower montane ecosystems in the western United States, and the western margin of eastern temperate forests. Here, a large fraction of the historically present trailing‐edge populations are at risk, despite lower absolute species counts. Together, these maps identify species‐rich climate risk hotspots and areas of concentrated relative risk to guide conservation planning.

### 3.2 Prioritizing Conservation Collections

To prioritize seed collections for assisted migration and long‐term genetic conservation, we used three criteria that capture complementary dimensions of risk and conservation value: (1) projected forest habitat loss, representing the likelihood of population extirpation; (2) climate change velocity, representing the geographic distances populations would need to migrate to remain within suitable climate, and thus the potential need for human intervention; and (3) the number of at‐risk trailing‐edge populations within each ecosystem, representing conservation value.

**FIGURE 2:**
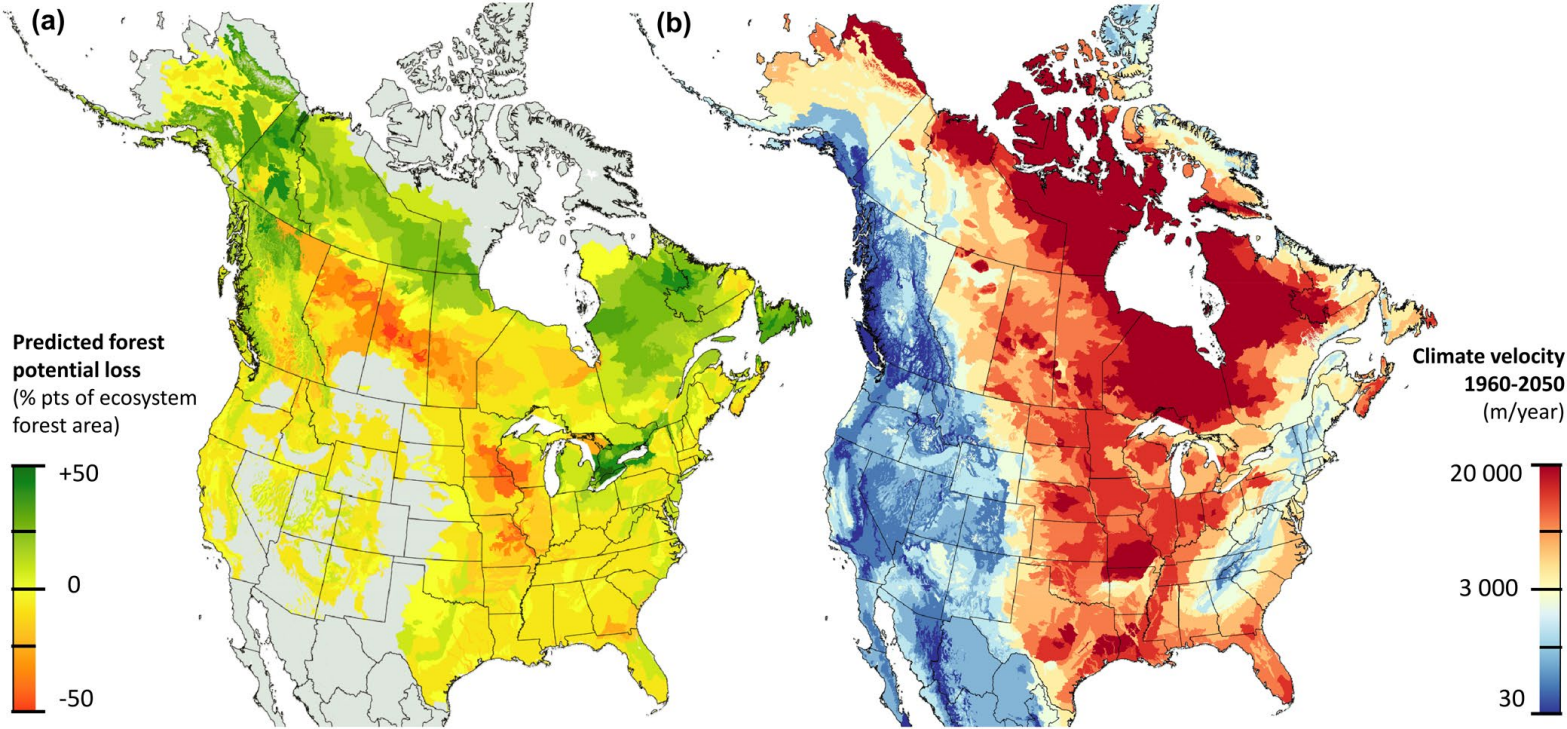
Climate‐related risk factors for prioritizing gene conservation collections. (a) Projected forest cover loss between 1960s and 2050s, indicating regions where climatic conditions may exceed the physiological limits of forest tree species. (b) Climate velocity (m/year), representing the distance populations must shift annually to remain within their historical climate envelope, and highlighting areas where natural migration may be insufficient.

A spatially explicit representation of projected habitat loss in units of percentage points (Figure 2a) suggests that forest declines will be concentrated in the southwestern boreal forest and the western margins of the eastern temperate forests. These projections are driven by future climate conditions that exceed all tree species' physiological tolerances and were calculated as the difference between reconstructed historical tree species frequencies and modelled future habitat across all 100 tree species included in the study (for details and visualization of future projections, refer to Appendix S2).

The second criterion, mapped 1960s–2050s climate velocity (Figure 2b), highlights that long‐distance migration of organisms to maintain historical climate conditions coincides with the interior plains of North America. For forested ecosystems, those previously identified as at risk due to loss of forest cover (i.e., southwestern boreal, and western portions of eastern temperate forests) also face high climate change velocity values (compare Figure 2a,b).

As the third criterion, conservation value, we used the number of trailing‐edge populations at risk (Figure 1a). Notably, the highest conservation value does not coincide with the greatest threats projected for the 2050s. Trailing‐edge populations in the Appalachian and Great Lakes regions either do not face climate habitats that are generally unsuitable for all tree species or require only moderate dispersal distances, with nearby suitable habitat available at higher elevation.

**FIGURE 3:**
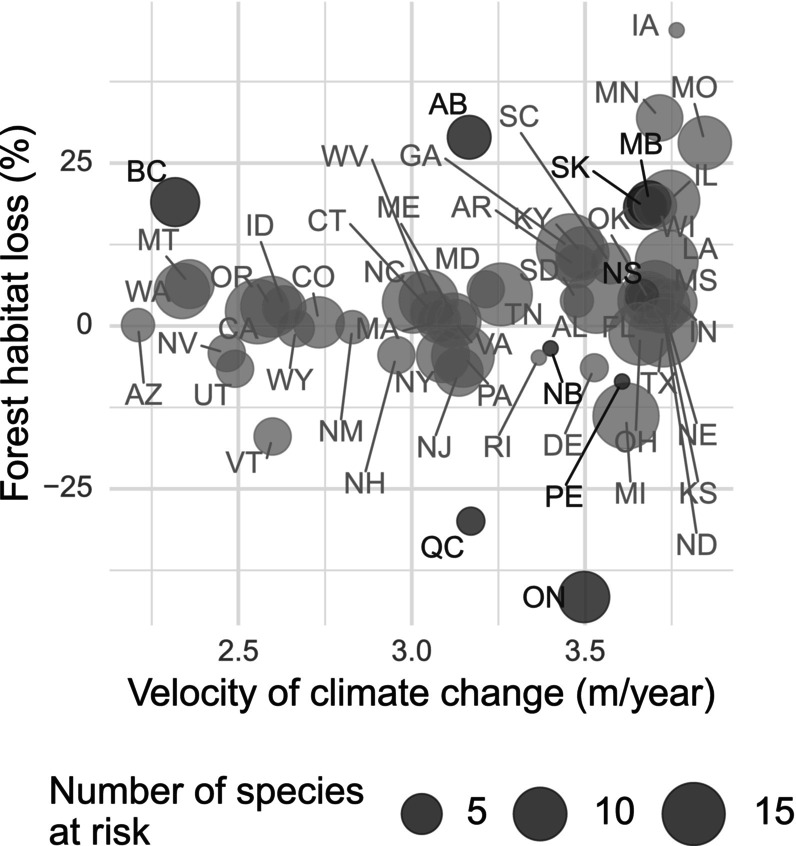
Near‐future climate risks for genetic diversity, aggregated by jurisdictions. States and provinces in the upper right face the largest projected forest cover loss by the 2050s (indicating fundamental niche limits for all species are exceeded on a portion of their land base) and the highest climate change velocity values (indicating the need for human intervention). The responsibility of jurisdictions with regard to conservation values is represented by the size of circles.

The three criteria can also be summarized at the jurisdictional level by aggregating projections across provinces and states (Figure 3). This provides an overview for resource allocation and policy planning, identifying jurisdictions where near‐future climate change may pose the most urgent and widespread threats to forest genetic resources. The combination of climate velocity and projected forest cover loss points to conservation priorities in the upper quadrant, with point size scaled by the number of species projected to experience trailing‐edge climate habitat loss, indicating conservation value.

### 3.3 Evaluating Recipients for in situ Conservation

To assess potential recipient sites for assisted migration and in situ conservation of trailing‐edge populations, we evaluated ecosystem demand for their potential to receive climate‐threatened populations and capacity for in situ conservation under 2050s climate conditions. Demand was defined as the number of climate‐threatened populations for which an ecosystem is projected to provide suitable habitat, while capacity was estimated as the area of IUCN‐categorized protected land within the ecosystem, adjusted for its projected forest cover potential under 2050s climate.

**FIGURE 4:**
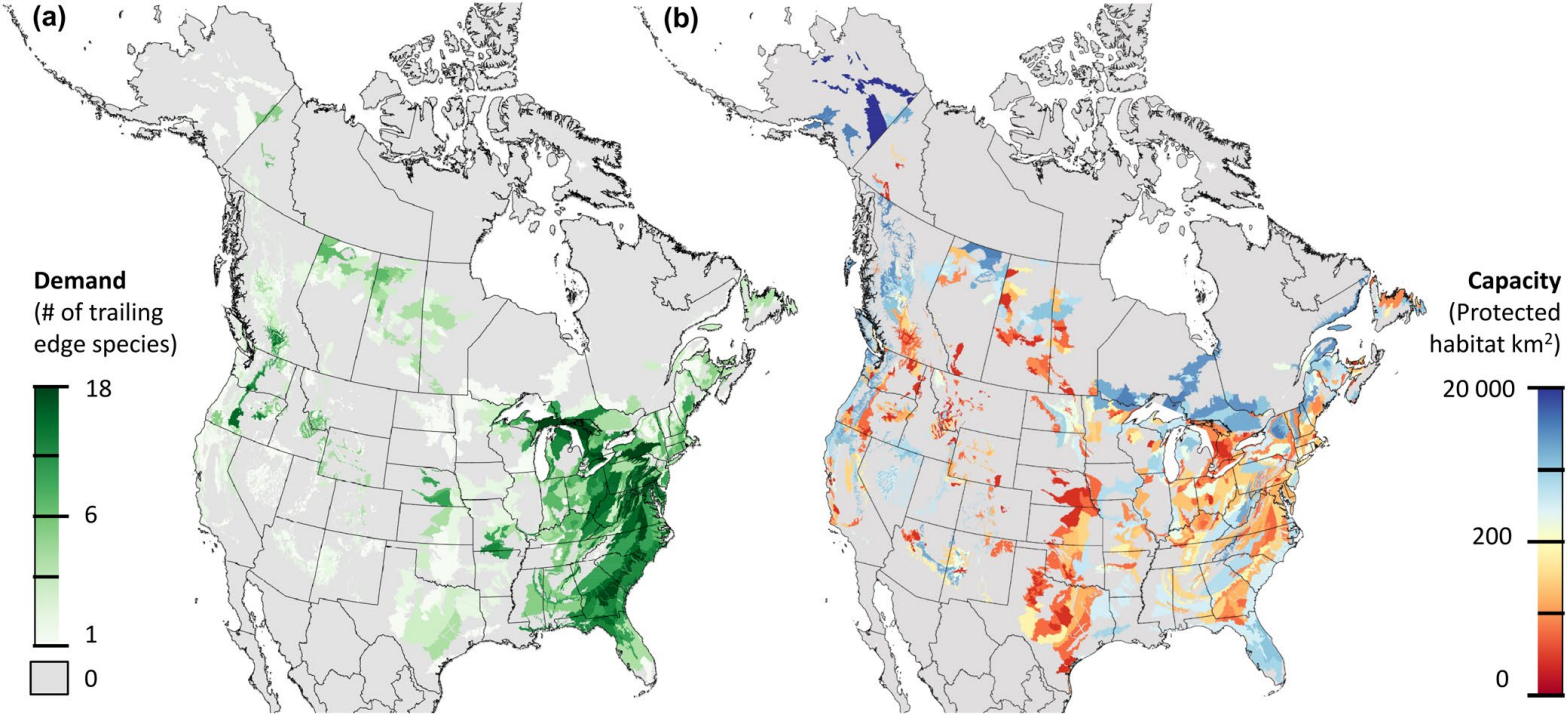
Projected demand and capacity for incoming assisted migration of trailing‐edge populations at risk. (a) Demand quantified as the number of species with climate‐threatened trailing‐edge populations for which the ecozone is projected to provide suitable climatic habitat under 2050s conditions. (b) Capacity for accepting assisted migration within ecosystems with demand. Capacity is measured as the projected area of protected forest (km^2^) under the 2050s forest cover scenarios. Ecozones with no demand have been excluded and are represented in grey.

Mapping projected demand across the study region revealed spatial patterns in future climate suitability for displaced populations (Figure 4a). High‐demand ecozones include the Great Lakes region, particularly southern Ontario and northern Minnesota, as well as the northern Appalachians, with concentrations in Pennsylvania, West Virginia, and Ohio. Smaller areas of concentration are found on the east coast in New Brunswick, providing plausible climate refugia with a substantial buffer due to oceanic influences. These areas are predicted to offer climate conditions analogous to the historical habitat of many trailing‐edge populations and may play a key role in maintaining genetic diversity through conservation plantings or restoration efforts.

Projected demand is generally well aligned with the amount of capacity, measured as climatically suitable habitat for trailing‐edge populations in protected areas (Figure 4b). This suggests that translocated populations could be well accommodated within the existing protected area network (blue shades). However, several ecozones, broadly scattered throughout western Canada and the United States, show high projected demand with comparatively limited capacity for in situ conservation within the current protected area network (orange shades).

**FIGURE 5:**
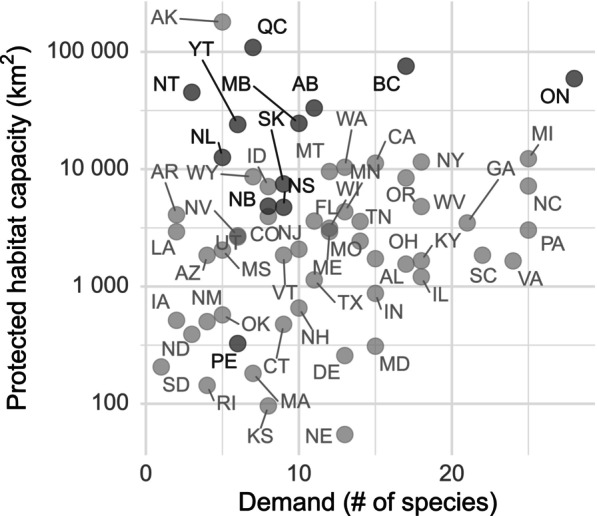
Jurisdictional summary of projected demand and capacity for assisted migration. Each province or state is plotted by the number of threatened species projected to find suitable climate habitat (demand) and the area of protected forest projected for the 2050s (capacity, in log_10_ km^2^). Jurisdictions in the upper right combine high demand with high capacity, suggesting strong conservation opportunities.

To support jurisdictional planning, we again aggregated demand and capacity by province and state (Figure 5). This summary helps identify jurisdictions with particularly strong potential to support future populations at risk and where policy or investment could maximize long‐term conservation benefits. Jurisdictions in the top right quadrant, such as Ontario, stand out as prime recipient jurisdictions for conservation‐focused assisted migration, having both high demand and high capacity. In contrast, jurisdictions to the center/lower right may benefit from additional protected areas to secure the value of in situ gene conservation efforts.

## 4 Discussion

### 4.1 Strategic Conservation of Trailing‐Edge Populations

The observed concentrations of trailing‐edge populations in the Appalachians, located in the states of North Carolina, Tennessee, and Virginia, coincide with a region of high species diversity where many important northeastern forest tree species have their southern distribution limits (Currie and Paquin 1987; Dexter et al. 2019; Hart et al. 2014). These areas provide cooler microclimates at higher elevations that allow northern species to persist farther south than they otherwise could. This includes forest tree species of major economic and ecological importance, such as red spruce ( *Picea rubens* ), sugar maple ( *Acer saccharum* ), and American beech ( *Fagus grandifolia* ). However, for rear‐edge populations of northern species that are located within temperate forest ecosystems, adaptive genetic variation to climate extremes should not automatically be assumed.

In a broader context, trailing‐edge populations of species that are located well within climate conditions that support forest ecosystems are not necessarily at imminent risk of extirpation. In the absence of a major disturbance, maladaptation to climate alone is unlikely to drive large‐scale mortality or contraction over the next few decades (Parks et al. 2019). In mountainous regions, such as the Appalachians and Pacific Northwest, elevational gradients offer microrefugia, allowing populations to persist or migrate over short distances to remain within suitable climates (Suggitt et al. 2018). Closer inspection of the hotspots of trailing‐edge populations identified within the Appalachian range reveals that they are primarily concentrated in lower elevations such as the northern shale valleys. In contrast, nearby higher elevation habitat exhibits low levels of species threat, suggesting that the climate threatened populations in the region can travel smaller distances to find suitable climatic habitat by migrating upslope (i.e., low climate velocities). Second, the high‐elevation populations of the region are not at immediate risk of climate‐related extirpation, but may be subject to long‐term decline due to competition from better adapted species.

Instead, we make a case to direct gene conservation priorities toward the flatter landscapes of the Midwest and southwestern boreal forest, where our analysis identified high climate velocity and high relative forest cover loss. Despite generally having fewer tree species overall, the combination of high velocity and habitat loss, compounded by high levels of land conversion or frequent climate‐driven disturbances, such as drought, fire, and pests, elevates the risk of population loss. Here, stochastic disturbance events can act as tipping points, extirpating already stressed populations before conservation interventions are in place (Seidl et al. 2017). For forest managers, the results provide direct guidance for prioritization. Figure 3 identifies priority jurisdictions, while Table S1 provides species‐level priorities and Table S2 provides ecosystem‐ and population‐level priorities to guide in situ conservation efforts within each jurisdiction.

### 4.2 Connecting Threatened Populations to Suitable Recipient Sites

Identifying recipient sites for assisted migration can be viewed as a logistical conservation challenge. However, when implemented through regular reforestation and ecosystem restoration activities, it is also a strategic opportunity to harness warm‐adapted genetic diversity to sustain forest health and productivity in a changing climate. Rather than viewing assisted migration as a last‐resort intervention, we propose reframing it as a forward‐looking strategy to re‐establish adaptive potential where it is most needed. The target regions for trailing‐edge populations identified in this study are not just passive recipients of displaced populations. They are staging grounds where potentially valuable genetic legacies of trailing‐edge populations can persist, evolve, and contribute to resilient future forests.

While many jurisdictions appear well‐positioned to receive incoming populations, macro‐climatic habitat suitability alone is no guarantee of success. Additional criteria are important to ensure the survival and integration of translocated genotypes. For example, some areas may offer climatically suitable habitat but lack appropriate soil substrates, disturbance regimes, or successional stages needed for successful establishment (Halofsky et al. 2020; Ni and Vellend 2024). Others may support forest ecosystems in principle, but are so fragmented by agriculture or development that landscape‐level connectivity becomes a limiting factor to future maintenance of genetic diversity and evolutionary potential (Parks et al. 2023). In this context, protected areas are valuable not just for their permanence but also for their capacity to offer relatively intact ecological templates where species interactions, nutrient cycles, and disturbance dynamics can proceed relatively unimpeded (Parks et al. 2023).

Potential ecological risks of translocation also need to be considered. Introducing genotypes outside their native context can potentially disrupt local ecosystem functions, facilitate hybridization, or unintentionally spread pests and pathogens (Winder et al. 2011). While these concerns are often raised in the context of non‐native species introductions, they also apply, albeit to a lesser extent, to assisted migration of native species or genotypes, alongside other risks such as outbreeding depression or loss of locally adapted diversity (Aitken and Whitlock 2013; Williams and Dumroese 2013). To mitigate these risks, seed transfers should prioritize genetic affinity and ecological fit: matching the climate envelope and shared biotic communities, soil types, and disturbance histories. Incorporating genetic screening, genomic vulnerability assessments, and common garden trials (Capblancq et al. 2020; Johnston et al. 2009; USDA Forest Service 2024) can help ensure that conservation translocations are ecologically sensitive and practically effective.

While the ecological rationale for assisted migration is gaining clarity as climate continues to change, the success of such programs hinges on social acceptance, institutional coordination, and policy frameworks. As previous studies have emphasized (Pedlar et al. 2012; Schwartz et al. 2012), public concerns over “tampering with nature,” uncertainties about long‐term impacts, or conflicting land‐use priorities can pose barriers to implementation. Our jurisdiction‐level results reveal that some of the most promising recipient areas, such as southern Ontario, Pennsylvania, or Ohio, are dominated by private landownership or heavily modified landscapes, where regulatory authority is diffused and competing interests can be expected (Ontario Ministry of Natural Resources 2021; USDA Forest Service 2021). In these contexts, partnerships with private landowners, conservation NGOs, and Indigenous communities will be critical. Incentive programs that align conservation with economic or cultural values, such as carbon offset credits, agroforestry schemes, or community seed banks, may be needed to move beyond the confines of public land and integrate assisted migration into broader land‐use systems.

Lastly, while our study provides a spatially explicit foundation for identifying where conservation resources could have the greatest immediate impact for trailing‐edge populations, prioritization is only the first step. Operationalizing assisted migration at meaningful scales requires sustained investments in seed collection, storage, propagation, and monitoring, all of which bring logistical challenges and knowledge gaps. For instance, the future suitability of recipient sites will depend not only on climate conditions, but also on future fire regimes, pest pressures, and land‐use change, which are more difficult to predict. Moreover, some of the trailing‐edge populations identified in this study may or may not represent unique ecotypes or harbor genetic traits of value. Investing in short‐ and long‐term common garden testing or genomic analysis could help to reduce assisted migration efforts that do not have adaptive benefits. In this sense, our spatial analysis is best viewed as a decision‐support tool that invites refinement as new ecological, genetic, and socio‐political information becomes available.

### 4.3 Applying the Conservation Framework: Uses and Limitations

To help identify target conservation sites for populations of concern, we developed a companion online tool: the Protected Area Selection Tool for North America (PAST‐NAm, https://tinyurl.com/past‐nam). It enables users to identify climatically suitable protected areas for any population of concern, including the trailing‐edge populations examined in this study, guided by the same multivariate climate matching approach at the ecosystem level as described in this study. A key assumption in this framework is that ecosystem delineations track climate and species communities closely. In many areas, this assumption holds well, especially in plains and lowland forests. However, in mountainous terrain, ecosystems often span steep elevation gradients or patchy microclimates. While PAST‐NAm introduces elevation bands to improve resolution, these do not yet align with species turnover thresholds, which may lead to erratic migration recommendations in topographically complex regions. In such cases, managers are advised to treat PAST‐NAm outputs as coarse indicators and refine selections with local knowledge.

Another caveat relates to the matching of future and historical climates. The tool uses 30‐year climate normals for both past (1960s, 1990s) and future projections (2020s, 2050s and 2080s), as these provide a more stable signal than decadal averages. However, observed historical climate trajectories sometimes diverge from ensemble‐based future projections in either magnitude or direction. For example, a location may have warmed faster than models anticipated, or become drier instead of wetter. Users should consider this divergence when interpreting results: if observed change exceeds projected trends, it may be appropriate to advance the time horizon (e.g., use 2080s projections instead of 2050s). In contrast, if observed change has lagged behind projections, a more conservative migration strategy may be warranted. Ultimately, adaptation efforts must align with observed climate trajectories, not with projections.

While PAST‐NAm helps guide species transfers based on climatic suitability, it does not account for non‐climatic ecological factors that are often critical to establishment. Rare or low‐frequency species may be habitat specialists, restricted to riparian zones, particular soil types, or unique disturbance regimes. For these, climate matching must be supplemented with habitat‐specific silvics knowledge or local field assessments, as is standard under current reforestation practices. Ecosystem delineations only provide broad guidelines as to which species should be considered for reforestation and ecosystem restoration, and forest managers must still make species choices for specific site conditions. The same approach is needed for assisted migration under climate change.

## 5 Conclusions

This study identifies where North American forests hold their most vulnerable yet potentially valuable genetic resources. Trailing‐edge populations at the warm or dry limits of species' ranges are concentrated in the Appalachians and Great Lakes, while the Midwest and southwestern boreal forests face the greatest risks from habitat loss and rapid climate displacement. These regional contrasts highlight the need for coordinated conservation that integrates genetic distinctiveness, climatic risk, and landscape connectivity. Our analysis supports a dual strategy for in situ conservation: conserving high‐value trailing‐edge populations in protected areas and incorporating assisted migration in regular reforestation or ecosystem restoration activities to utilize their adaptive potential under climate change. The accompanying PAST‐NAm tool enables these actions by linking at‐risk populations to suitable climate habitats in the future, as well as climate refugia in protected areas. Together, the approach offers a practical framework to inform efforts aimed at maintaining the evolutionary potential and ecological resilience of North American forests under climate change.

## Author Contributions

**Genevieve Dorrell:** investigation, data curation, formal analysis, writing – review and editing. **Nicholas Boyce:** conceptualization, investigation, writing – original draft, methodology, visualization, formal analysis. **Scott E. Nielsen:** conceptualization, supervision, writing – review and editing. **Andreas Hamann:** conceptualization, investigation, funding acquisition, methodology, writing – review and editing, supervision, project administration, software.

## Funding

This work was supported by the Natural Sciences and Engineering Research Council of Canada, RGPIN‐2020‐04189, ALLRP 583409‐23 and Mitacs.

## Conflicts of Interest

The authors declare no conflicts of interest.

## Supporting information


**Appendix S1:** Methodological supplement on reconstructing natural forest cover.
**Appendix S2:** Methodological supplement on identifying trailing edge populations.
**Figure S1:** Ecosystem averages of forest coverage based on MODIS vegetation continuous field data (left), and with agricultural and urban areas backfilled according to the most probable land cover class (right).
**Figure S2:** Climatic habitat supportive of different biomes for the 1960s baseline historic period (left) and projected 2050s climate (right). The predictions are based on a majority vote of biome types from the 5 best‐matching level‐4 ecosystems.
**Figure S3:** Climatic habitat supportive forest cover, assuming no human development for the 1960s baseline historic period (left) and projected 2050s climate (right).
**Figure S4:** Examples of trailing edge tree populations at risk of climate habitat loss by the 2050s (red colors).
**Table S1:** List of potentially valuable trailing edge populations by species.
**Table S2:** List of ecosystems with potentially valuable trailing edge populations for the most common 100 North American tree species.

## Data Availability

All data used in this study are publicly available. Canadian national forest inventory data https://nfi.nfis.org/en were accessed at: https://saforah2.nfis.org/CSWClient (search term: “forest attributes knn”). United States forest inventory data are available at: https://www.fs.usda.gov/rds/archive/catalog/RDS‐2013‐0013. Climate data are accessible at http://tinyurl.com/ClimateNA. Protected area data are available from the World Database on Protected Areas: https://www.protectedplanet.net.
