# Supplementary material for: Prioritizing Conservation of Trailing‐Edge Populations for Future Climate‐Resilient Forests
Source: Glob Chang Biol. 2026 Jun 25;32(6):e70971. doi: 10.1111/gcb.70971 (PMC13296265; doi:10.1111/gcb.70971)
Supplement: Supplementary file 1 — Appendix S1: Methodological supplement on reconstructing natural forest cover. Appendix S2: Methodological supplement on identifying trailing edge populations. Figure S1: Ecosystem averages of forest coverage based on MODIS vegetation continuous field data (left), and with agricultural and urban areas backfilled according to the most probable land cover class (right). Figure S2: Climatic habitat supportive of different biomes for the 1960s baseline historic period (left) and projected 2050s climate (right). The predictions are based on a majority vote of biome types from the 5 best‐matching level‐4 ecosystems. Figure S3: Climatic habitat supportive forest cover, assuming no human development for the 1960s baseline historic period (left) and projected 2050s climate (right). Figure S4: Examples of trailing edge tree populations at risk of climate habitat loss by the 2050s (red colors). Table S1: List of potentially valuable trailing edge populations by species. Table S2: List of ecosystems with potentially valuable trailing edge populations for the most common 100 North American tree species. [file GCB-32-e70971-s001.pdf]

Supplementary information for the article:

# Prioritizing conservation of trailing-edge populations for future climate-resilient forests

Nicholas Boyce*, Andreas Hamann, Genevieve Dorrell, & Scott E. Nielsen

Department of Renewable Resources
Faculty of Agricultural, Life, and Environmental Sciences
University of Alberta
751 General Services Building
Edmonton, AB, Canada, T6G 2H1

* Corresponding author's contact information: nboyce@ualberta.ca

Content:

**Appendix S1.** Methodological supplement on reconstructing natural forest cover

**Appendix S2.** Methodological supplement on identifying trailing edge populations

**Figure S1.** Ecosystem averages of forest coverage based on MODIS vegetation continuous field data (left), and with agricultural and urban areas backfilled according to the most probable land cover class (right).

**Figure S2.** Climatic habitat supportive of different biomes for the 1960s baseline historic period (left) and projected 2050s climate (right). The predictions are based on a majority vote of biome types from the 5 best-matching level-4 ecosystems.

**Figure S3.** Climatic habitat supportive forest cover, assuming no human development for the 1960s baseline historic period (left) and projected 2050s climate (right).

**Figure S4.** Examples of trailing edge tree populations at risk of climate habitat loss by the 2050s (red colors).

**Table S1.** List of potentially valuable trailing edge populations by species.

**Table S2.** List of ecosystems with potentially valuable trailing edge populations for the most common 100 North American tree species.

## Appendix S1. Methodological supplement on reconstructing natural forest cover

To characterize available climate habitat in each ecosystem for each forest tree species, we estimated the proportion of land within each ecosystem that could support forest cover under natural conditions. This estimate serves two purposes: (1) the estimate is the basis for scaling species frequencies derived from plot and inventory data, so that total species abundance, combined with non-forested areas, would sum to 100% of the land area within each ecosystem; (2) the estimates were used to inform predictions for future forest cover as a potential risk factor, where predictions of forest cover loss can be interpreted as exceeding the climatic tolerance limits of all forest tree species (thereby also serving as a proxy for exceeding the fundamental niche space of all tree species included in the study).

The initial estimate of forest cover was derived from the MODIS Vegetation Continuous Fields (VCF) product, MOD44B Version 6 (DiMiceli et al. 2021), which provides a global fractional estimate of tree canopy cover at 250 m spatial resolution. Because substantial portions of potential forest land have been converted to agriculture and urban development, we generated an additional estimate of potential natural forest cover to more accurately reflect the area suitable for tree species in the absence of human disturbance. For this purpose, we used a corresponding 250m MODIS land cover classification product (CEC 2005).

A deep neural network classifier was trained to replace the agriculture and urban classes with the most probable natural land cover class based on the location's annual climate and topography. The neural network employed for land cover classification was a feed-forward deep learning model designed to predict 17 natural land cover classes, excluding water, agriculture, and urban classes from the training data set, but including the locations of agriculture and urban classes as prediction targets to be classified. The model was trained on 11 bioclimatic variables and 16 topographic predictors, including terrain indices such as topographic position and convergence, aspect components (northness, southness), exposure, and proximity to water bodies (lakes, rivers, and oceans), following the methodology described in detail by Namiiro et al. (2005). We implemented the model using the Keras package for R, with Google's TensorFlow v2.10.1 machine learning platform for Python 3.9 as the computational backend, using the following feed-forward architecture:

```
model = keras_model_sequential() %>%
  layer_dense(units = 4096, activation = 'relu',
              input_shape = ncol(train_x)) %>%
  layer_dense(units = 2048, activation = 'relu') %>%
  layer_dense(units = 1024, activation = 'relu') %>%
  layer_dense(units = 512, activation = 'relu') %>%
  layer_dense(units = 256, activation = 'relu') %>%
  layer_dense(units = 128, activation = 'relu') %>%
  layer_dense(units = 64, activation = 'relu') %>%
  layer_dense(units = 32, activation = 'relu') %>%
  layer_dense(units = 17, activation = 'softmax')

model %>% compile(
  loss = 'categorical_crossentropy',
  optimizer = optimizer_adam(),
  metrics = 'accuracy'
)
```

This process yielded a reconstruction of potential natural land cover. By summing the proportion of pixels predicted to be forest cover within each ecosystem, we obtained an expected proportion of forested land cover that could be used to scale relative tree species frequencies and predict changes to climate habitat suitable for tree cover under climate change projections, independent of human disturbance levels (Figure S1).

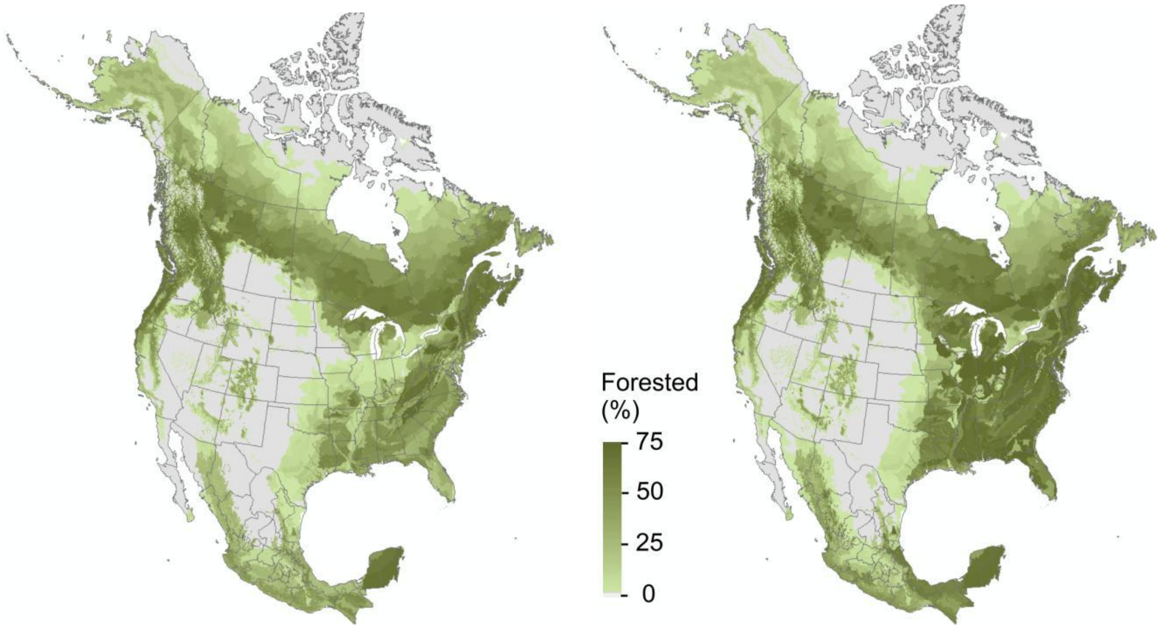


**Figure S1.** Ecosystem averages of forest coverage based on MODIS vegetation continuous field data (left), and with agricultural and urban areas backfilled according to the most probable land cover class (right).

## References (Appendix S1)

CEC. 2005. The North American Land Change Monitoring System. Land Cover, 2005 (MODIS, 250m) URL: https://www.cec.org/north-american-environmental-atlas/land-cover-2005-modis-250m/, accessed July 12, 2024. Commission for Environmental Cooperation, Montreal, Canada.

DiMiceli, C., J. Townshend, M. Carroll, and R. Sohlberg. 2021. Evolution of the representation of global vegetation by vegetation continuous fields. *Remote Sensing of Environment* **254**: e112271.

Namiiro, S., A. Hamann, T. Wang, D. Castellanos-Acuña, and C. R. Mahony. 2025. A high-resolution database of historical and future climate for Africa developed with deep neural networks. *Scientific Data* **11**:1278.

**Appendix S2. Methodological supplement on identifying trailing edge populations**

To make predictions of changes to suitable climate habitat for forest cover in general, as well as for individual tree species, we used an ecosystem-based climate matching approach, conceptually similar to those used for example by Hamann and Wang (2006), Gray et al. (2011) or Rehfeldt et al. (2012). This approach relies on ecosystem delineations as modeling units.

We used the 1960s climate (1951–1980) as the baseline reference and matched it to the projected 2050s climate (2041–2070) using a standardized Euclidean distance matrix based on 11 selected bioclimatic variables following the methodology described in Gray et al. (2011). While the finest Level-4 ecosystem delineations were used for climate matching, a visualization colorized by major ecosystem class (Level-1) provides an initial high-level assessment of the spatial pattern and severity of shifts in climate habitat relevant to forest ecosystems (Figure S2).

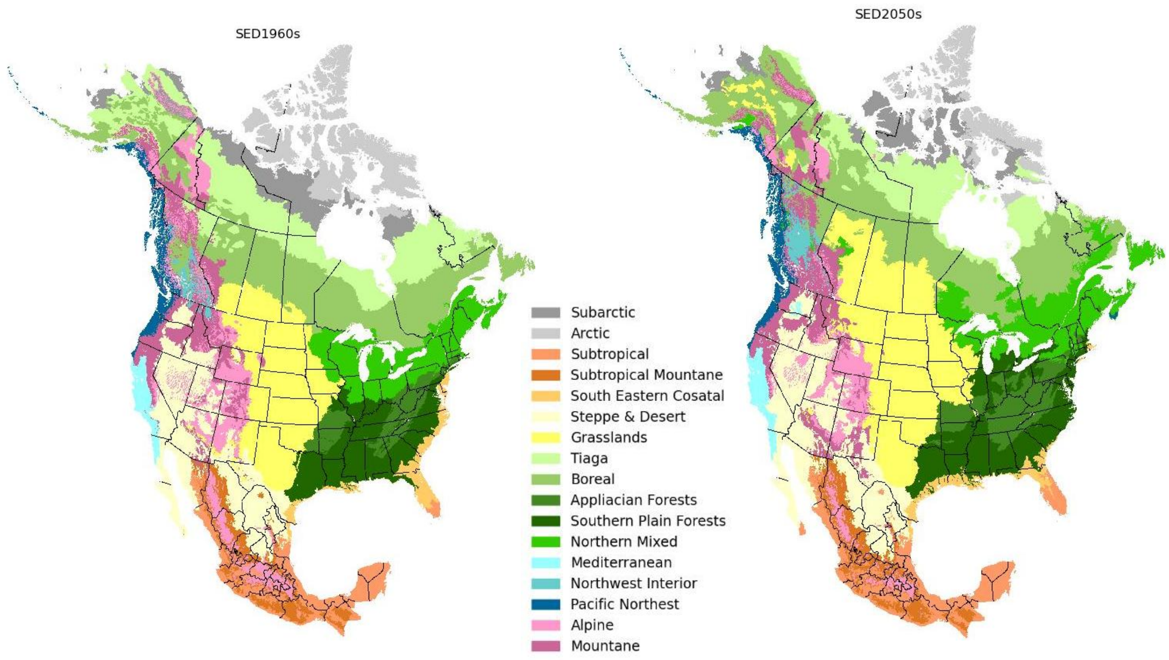


**Figure S2.** Climatic habitat supportive of different biomes for the 1960s baseline historic period (left) and projected 2050s climate (right). The predictions are based on a majority vote of biome types from the 5 best-matching level-4 ecosystems.

Similarly, projected Level-4 ecosystem classes can represent any ecosystem attribute, such as the expected frequency of individual species or the total expected proportion of forested habitat (Figure S3), excluding human development (see Supplement 1).

On the same basis, trailing-edge populations were inferred by habitat loss under projected climate change. To be included in the analysis, we also required that the candidate population was within 200km of the species' historical range based on spatially buffered Little (1971) range maps. This dual criterion helped exclude spurious trailing edge population

identifications resulting from either taxonomic misidentification or planted/introduced occurrences outside the native species distribution (Figure S4).

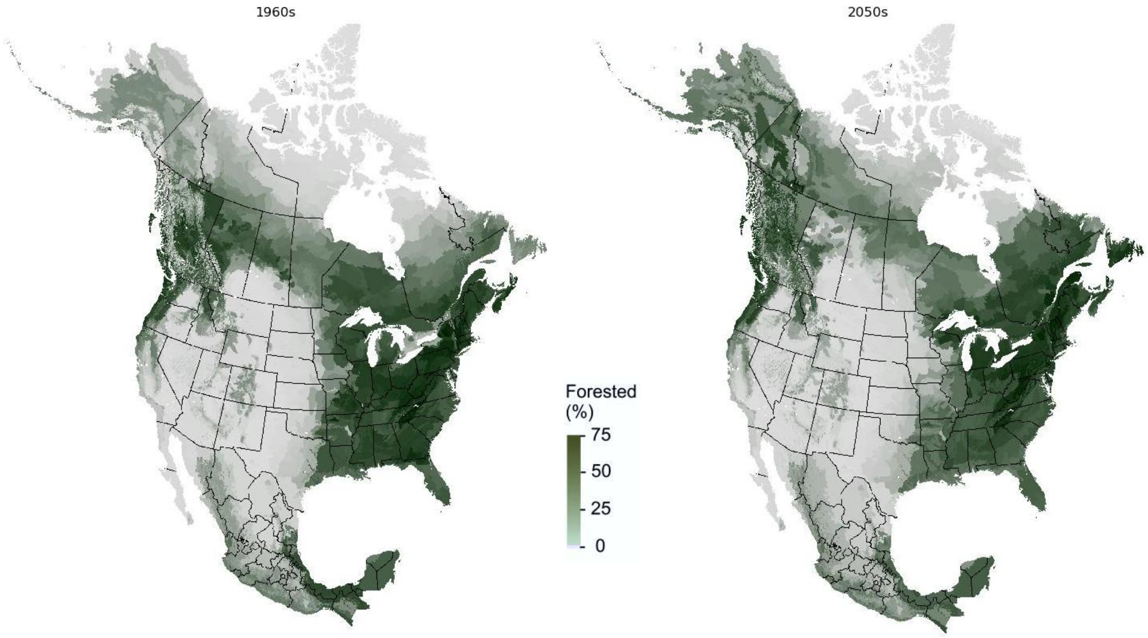


**Figure S3.** Climatic habitat supportive forest cover, assuming no human development for the 1960s baseline historic period (left) and projected 2050s climate (right). The projection is used as a risk factor to evaluate the need for gene conservation in the short term due to high risk of population extirpation. The predictions are based on the average reconstructed natural forest cover from the 5 best-matching level-4 ecosystems.

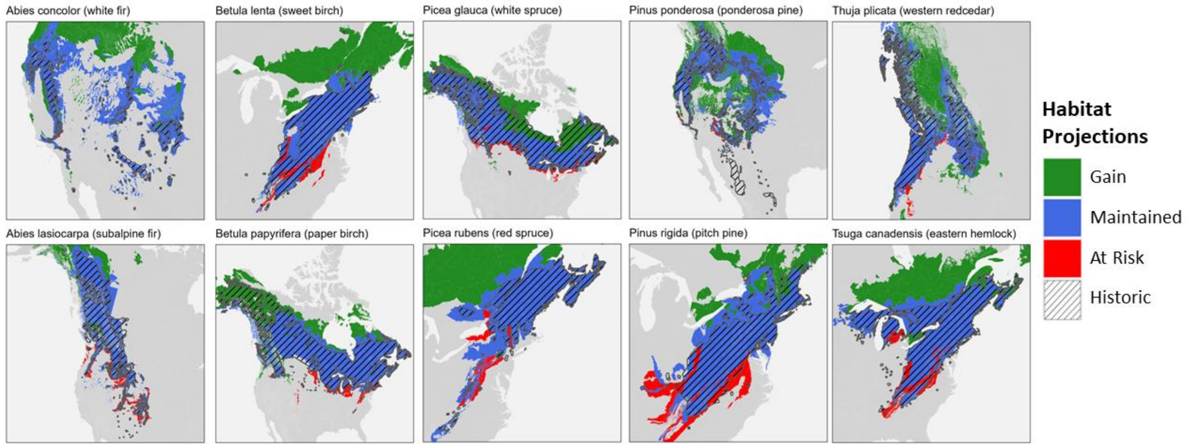


**Figure S4.** Examples of trailing edge tree populations at risk of climate habitat loss by the 2050s (red colors). As an additional criterion to qualify as a trailing edge population, the population needed to be within 200 km of the historical species range as mapped by Little (1971) to reduce spurious trailing edge population identifications resulting from either taxonomic misidentification or planted/introduced occurrences.

**References (Appendix S2)**

Gray, L. K., Gylander, T., Mbogga, M. S., Chen, P. and Hamann, A. (2011). Assisted migration to address climate change: recommendations for aspen reforestation in western Canada. Ecological Applications. 21: 1591–1603.

Hamann, A., & Wang, T. (2006). Potential effects of climate change on ecosystem and tree species distribution in British Columbia. Ecology, 87(11), 2773–2786.

Little, E. L., 1971. Atlas of United States Trees, Volume 1: Conifers and Important Hardwoods. U.S. Department of Agriculture, Miscellaneous Publication 1146. Washington, D.C.

Rehfeldt, G. E., Crookston, N. L., Sáenz-Romero, C., & Campbell, E. M. (2012). North American vegetation model for land-use planning in a changing climate: A solution to large classification problems. Ecological Applications, 22(1), 119–141.

**Table S1.** List of potentially valuable trailing edge populations by species. Included are the 5 warmest (highest mean annual temperature) and the 5 driest (highest climate moisture deficit) ecosystem codes (EcoID) where trailing edge populations were identified. Ecosystem codes can be cross-referenced with Table S2. N indicates the total number of ecosystems with trailing edge populations, which may be zero if climate change projections did not indicate potential climate habitat loss at the species' warm or dry margin.

| SpecID | Species name | N | Warmest (EcoID) | Driest (EcoID) |
|---|---|---|---|---|
| abieamab | Pacific silver fir (*Abies amabilis*) | 8 | 1268, 1279, 1202, 1206, 1281 | 1281, 1389, 1211, 1054, 1268 |
| abiebals | balsam fir (*Abies balsamea*) | 46 | 1630, 1498, 1412, 1580, 1428 | 1316, 1580, 1571, 1547, 528 |
| abieconc | white fir (*Abies concolor*) | 1 | 1843 | 1843 |
| abiegran | grand fir (*Abies grandis*) | 6 | 1570, 1446, 1586, 1422, 1212 | 1570, 1422, 1212, 1361, 1446 |
| abielasi | subalpine fir (*Abies lasiocarpa*) | 27 | 1207, 1209, 1281, 1327, 1199 | 1682, 1499, 1877, 1775, 1658 |
| acermacr | bigleaf maple (*Acer macrophyllum*) | 0 | NA | NA |
| acernegu | boxelder (*Acer negundo*) | 11 | 1719, 1648, 1906, 2236, 1983 | 1906, 2028, 1865, 1983, 1957 |
| acerpens | striped maple (*Acer pensylvanicum*) | 15 | 2015, 1871, 1932, 1973, 1807 | 1871, 1807, 1878, 1678, 1870 |
| acerrubr | red maple (*Acer rubrum*) | 0 | NA | NA |
| acersacc | silver maple (*Acer saccharinum*) | 7 | 2131, 2120, 2114, 2113, 2109 | 1790, 2048, 2109, 2113, 2114 |
| acersacr | sugar maple (*Acer saccharum*) | 28 | 1984, 1972, 2006, 1970, 1965 | 1968, 1984, 1990, 1972, 1902 |
| acerspic | mountain maple (*Acer spicatum*) | 18 | 2250, 1871, 1807, 1755, 1765 | 1871, 2250, 1807, 1678, 1571 |
| alnurubr | red alder (*Alnus rubra*) | 1 | 1555 | 1555 |
| arbumenz | Pacific madrone (*Arbutus menziesii*) | 0 | NA | NA |
| betualle | yellow birch (*Betula alleghaniensis*) | 20 | 1870, 1937, 2003, 1878, 1884 | 1688, 1878, 1635, 1601, 1678 |
| betulent | sweet birch (*Betula lenta*) | 5 | 1859, 1871, 1807, 1878, 1805 | 1871, 1805, 1859, 1807, 1878 |
| betunigr | river birch (*Betula nigra*) | 6 | 2124, 2116, 2091, 2129, 2101 | 2101, 2124, 2116, 2091, 2112 |
| betupapy | paper birch (*Betula papyrifera*) | 58 | 1905, 1641, 1583, 1673, 1528 | 1277, 1295, 1344, 1267, 1318 |
| betupopu | gray birch (*Betula populifolia*) | 12 | 1689, 1696, 1615, 1663, 1674 | 1678, 1673, 1674, 1556, 1479 |
| carpcaro | American hornbeam (*Carpinus caroliniana*) | 1 | 2048 | 2048 |
| carycord | bitternut hickory (*Carya cordiformis*) | 2 | 2076, 1947 | 1947, 2076 |
| caryglab | pignut hickory (*Carya glabra*) | 15 | 2120, 2116, 2122, 2111, 2091 | 2088, 2076, 1968, 2125, 2109 |
| caryilli | pecan (*Carya illinoensis*) | 0 | NA | NA |
| caryovat | shagbark hickory (*Carya ovata*) | 2 | 2076, 2048 | 2048, 2076 |
| chamnoot | Alaska cedar (*Chamaecyparis nootkatensis*) | 4 | 1202, 1050, 1248, 1211 | 1211, 1248, 1050, 1202 |
| fagugran | American beech (*Fagus grandifolia*) | 9 | 2116, 2129, 2125, 2076, 2112 | 2076, 1902, 1883, 2125, 1889 |
| fraxamer | white ash (*Fraxinus americana*) | 5 | 2118, 2135, 2131, 2091, 2051 | 2118, 2091, 2051, 2131, 2135 |
| fraxnigr | black ash (*Fraxinus nigra*) | 32 | 1757, 1836, 1830, 1770, 1722 | 1741, 1688, 1635, 1601, 1678 |
| fraxpenn | green ash (*Fraxinus pennsylvanica*) | 3 | 2142, 1908, 1699 | 2142, 1699, 1908 |
| gledtria | honeylocust (*Gleditsia triacanthos*) | 3 | 2110, 2123, 1908 | 2110, 2123, 1908 |
| juglcine | butternut (*Juglans cinerea*) | 25 | 1926, 2043, 2042, 1984, 1939 | 2042, 2043, 1968, 1984, 1990 |
| juglnigr | black walnut (*Juglans nigra*) | 15 | 2110, 2123, 2125, 2076, 2100 | 2119, 1959, 2106, 2126, 2110 |
| junivirg | eastern redcedar (*Juniperus virginiana*) | 7 | 2100, 2112, 2109, 1992, 1959 | 1794, 1959, 1992, 1908, 2109 |
| larilari | tamarack (*Larix laricina*) | 55 | 1645, 1673, 1741, 1633, 1688 | 549, 1741, 1688, 1635, 557 |
| larilyal | subalpine larch (*Larix lyallii*) | 3 | 1231, 1303, 1060 | 1231, 1303, 1060 |
| lariocci | western larch (*Larix occidentalis*) | 9 | 1285, 1281, 1267, 1318, 1361 | 1361, 1353, 1313, 1285, 1281 |
| liqustyr | sweetgum (*Liquidambar styraciflua*) | 0 | NA | NA |
| lirituli | yellow poplar (*Liriodendron tulipifera*) | 11 | 2120, 2116, 2122, 2091, 2129 | 2076, 2021, 1984, 2125, 2102 |
| nyssaqua | water tupelo (*Nyssa aquatica*) | 1 | 2048 | 2048 |
| nysssylv | black tupelo, blackgum (*Nyssa sylvatica*) | 6 | 2118, 2124, 2127, 2110, 2088 | 2110, 2048, 2088, 2124, 2118 |
| ostrvirg | eastern hophornbeam (*Ostrya virginiana*) | 5 | 1700, 1649, 1654, 1686, 1539 | 1686, 1539, 1654, 1649, 1700 |
| piceenge | Engelmann spruce (*Picea engelmannii*) | 10 | 1297, 1281, 1267, 1447, 1775 | 1499, 1775, 1658, 1413, 1313 |
| piceglau | white spruce (*Picea glauca*) | 122 | 1635, 1625, 1479, 1509, 1521 | 1053, 1019, 998, 1075, 1056 |
| picemari | black spruce (*Picea mariana*) | 57 | 830, 1553, 1354, 1547, 1572 | 970, 745, 756, 978, 539 |
| picerube | red spruce (*Picea rubens*) | 6 | 1678, 1841, 1673, 1498, 1479 | 1841, 1678, 1673, 808, 1498 |
| picesitc | Sitka spruce (*Picea sitchensis*) | 0 | NA | NA |

| SpecID | Species name | N | Warmest (EcoID) | Driest (EcoID) |
|---|---|---|---|---|
| pinualbi | whitebark pine (*Pinus albicaulis*) | 18 | 604, 1465, 1281, 1201, 1606 | 1598, 1460, 1593, 1281, 1454 |
| pinubank | jack pine (*Pinus banksiana*) | 47 | 1732, 1717, 1677, 1720, 1640 | 549, 745, 1601, 539, 1580 |
| pinucont | lodgepole pine (*Pinus contorta*) | 18 | 1605, 1050, 1297, 1281, 1389 | 1438, 1485, 1499, 1459, 1495 |
| pinuechi | shortleaf pine (*Pinus echinata*) | 5 | 2135, 2120, 2116, 2101, 2051 | 2101, 2116, 2120, 2051, 2135 |
| pinuelli | slash pine (*Pinus elliottii*) | 0 | NA | NA |
| pinulamb | sugar pine (*Pinus lambertiana*) | 1 | 1852 | 1852 |
| pinumont | western white pine (*Pinus monticola*) | 3 | 1679, 1438, 1642 | 1679, 1642, 1438 |
| pinupalu | longleaf pine (*Pinus palustris*) | 0 | NA | NA |
| pinupond | ponderosa pine (*Pinus ponderosa*) | 6 | 1921, 1953, 1808, 1719, 1876 | 1921, 2024, 1808, 1953, 1876 |
| pinuresi | red pine (*Pinus resinosa*) | 2 | 1678, 1673 | 1678, 1673 |
| pinurigi | pitch pine (*Pinus rigida*) | 12 | 2015, 1859, 1871, 1936, 1826 | 1889, 1871, 1930, 1946, 1826 |
| pinustrb | eastern white pine (*Pinus strobus*) | 20 | 2015, 2250, 2019, 2038, 1871 | 1871, 2250, 2019, 1805, 1807 |
| pinutaed | loblolly pine (*Pinus taeda*) | 2 | 2118, 2124 | 2124, 2118 |
| pinuvirg | Virginia pine (*Pinus virginiana*) | 5 | 2046, 2082, 1939, 2008, 2034 | 2046, 2082, 2008, 1939, 2034 |
| platocci | American sycamore (*Platanus occidentalis*) | 1 | 2126 | 2126 |
| popubals | balsam poplar (*Populus balsamifera*) | 61 | 1732, 1684, 1688, 1707, 1635 | 1349, 1095, 1304, 645, 652 |
| popudelt | eastern cottonwood (*Populus deltoides*) | 5 | 2000, 1959, 1908, 1957, 1865 | 1865, 1957, 1959, 2000, 1908 |
| popugran | bigtooth aspen (*Populus grandidentata*) | 39 | 1776, 1817, 1826, 1946, 1796 | 1889, 1840, 1850, 1842, 1930 |
| poputrem | quaking aspen (*Populus tremuloides*) | 79 | 1812, 1805, 1674, 1425, 1765 | 1564, 1607, 1646, 1425, 1429 |
| poputric | black cottonwood (*Populus trichocarpa*) | 5 | 1679, 1607, 1759, 1459, 1499 | 1607, 1679, 1759, 1499, 1459 |
| prunpens | pin cherry (*Prunus pensylvanica*) | 28 | 1674, 1645, 1641, 1662, 1732 | 1332, 1348, 1304, 1315, 1310 |
| prunsero | black cherry (*Prunus serotina*) | 3 | 2137, 2118, 2126 | 2126, 2137, 2118 |
| pseumenz | Douglas fir (*Pseudotsuga menziesii*) | 2 | 2060, 1906 | 1906, 2060 |
| queralba | white oak (*Quercus alba*) | 5 | 2116, 2129, 2101, 2100, 2112 | 2101, 2100, 2116, 2112, 2129 |
| querbico | swamp white oak (*Quercus bicolor*) | 16 | 1970, 1965, 1871, 2249, 1936 | 1990, 1902, 1883, 1898, 1871 |
| quercocc | scarlet oak (*Quercus coccinea*) | 18 | 2083, 2007, 2089, 1926, 1977 | 2083, 2094, 2042, 2043, 1968 |
| querfalc | southern red oak (*Quercus falcata*) | 4 | 2124, 2135, 2120, 2122 | 2124, 2120, 2122, 2135 |
| querlaur | laurel oak (*Quercus laurifolia*) | 0 | NA | NA |
| querlyra | overcup oak (*Quercus lyrata*) | 0 | NA | NA |
| quermacr | bur oak (*Quercus macrocarpa*) | 3 | 2076, 1908, 2236 | 2236, 1908, 2076 |
| quermich | swamp chestnut oak (*Quercus michauxii*) | 1 | 2048 | 2048 |
| quermueh | chinkapin oak (*Quercus muehlenbergii*) | 2 | 1992, 1996 | 1992, 1996 |
| quernigr | water oak (*Quercus nigra*) | 0 | NA | NA |
| querpalu | pin oak (*Quercus palustris*) | 12 | 2021, 1984, 2044, 2036, 2039 | 2036, 2021, 2039, 1984, 2011 |
| querphel | willow oak (*Quercus phellos*) | 0 | NA | NA |
| querprin | chestnut oak (*Quercus prinus*) | 9 | 2083, 2233, 2094, 1984, 2046 | 2083, 2094, 1984, 2046, 2082 |
| querrubr | northern red oak (*Quercus rubra*) | 21 | 2043, 2042, 2094, 1914, 1984 | 2036, 2094, 2042, 2043, 2039 |
| quershum | Shumard oak (*Quercus shumardii*) | 0 | NA | NA |
| querstel | post oak (*Quercus stellata*) | 3 | 2124, 2135, 2120 | 2124, 2120, 2135 |
| quervelu | black oak (*Quercus velutina*) | 11 | 2128, 2109, 2090, 2104, 2066 | 2085, 2066, 2037, 2090, 2079 |
| quervirg | live oak (*Quercus virginiana*) | 0 | NA | NA |
| robipseu | black locust (*Robinia pseudoacacia*) | 1 | 2039 | 2039 |
| salinigr | black willow (*Salix nigra*) | 3 | 2041, 1959, 1794 | 2041, 1794, 1959 |
| sequsemp | redwood (*Sequoia sempervirens*) | 2 | 1808, 1786 | 1808, 1786 |
| sorbamer | American mountain ash (*Sorbus americana*) | 18 | 1870, 1891, 1755, 1756, 1678 | 1678, 1870, 1891, 1673, 1483 |
| taxodist | baldcypress (*Taxodium distichum*) | 1 | 2048 | 2048 |
| thujocci | northern white cedar (*Thuja occidentalis*) | 12 | 1732, 1684, 1635, 1677, 1556 | 1635, 1601, 1580, 1625, 1684 |
| thujplic | western redcedar (*Thuja plicata*) | 6 | 1422, 1389, 1212, 1277, 1313 | 1422, 1212, 1313, 1277, 1389 |
| tiliamer | American basswood (*Tilia americana*) | 17 | 1984, 2033, 2005, 2249, 1968 | 1558, 1304, 1968, 1984, 1325 |
| tsugcana | eastern hemlock (*Tsuga canadensis*) | 18 | 2082, 2064, 2019, 2038, 1871 | 2082, 1871, 2064, 2019, 1805 |

| SpecID | Species name | N | Warmest (EcoID) | Driest (EcoID) |
|---|---|---|---|---|
| tsughete | western hemlock (*Tsuga heterophylla*) | 6 | 1470, 1586, 1281, 1389, 1250 | 1470, 1313, 1281, 1389, 1250 |
| tsugmert | mountain hemlock (*Tsuga mertensiana*) | 4 | 1281, 1313, 1598, 1373 | 1598, 1313, 1281, 1373 |
| ulmuamer | American elm (*Ulmus americana*) | 0 | NA | NA |
| ulmurubr | slippery elm (*Ulmus rubra*) | 1 | 1790 | 1790 |

**Table S2.** List of ecosystems with potentially valuable trailing edge populations for the most common 100 North American tree species. Only ecosystems with species populations that were listed in Table S1 are included. Velocity of climate change (m/year) represents a risk factor to prioritize the need for human intervention (assisted migration). Habitat denotes a projected change of climate habitat suitable for forested land cover in an ecosystem (in units of percentage points). Negative values can be interpreted as a risk of direct climate impacts, where projected climate habitat exceeds the tolerances of all tree species included in this study. Positive values indicated a net gain in suitable climate habitat for forest trees, which can be interpreted as a less immediate threat to trailing edge populations, of potentially being outcompeted by other species in the long-term.

| State | EcoID | Ecosystem Name | Lat | Long | Velocity | Habitat | Species with potentially valuable populations |
|---|---|---|---|---|---|---|---|
| AL | 2082 | Shale Hills | 33.64 | -87.3 | 2951 | 6 | pinuvirg, querprin, tsugcana |
| AL | 2112 | Southern Pine Plains and Hills | 31 | -88.3 | 5184 | 15 | betunigr, fagugran, junivirg, queralba |
| AL | 2046 | Fall Line Hills | 33.33 | -87.5 | 3141 | 5 | pinuvirg, querprin |
| AL | 2019 | Sequatchie Valley | 34.81 | -85.9 | 1306 | -4 | pinustrb, tsugcana |
| AL | 2038 | Southern Table Plateaus | 34.32 | -86.2 | 2315 | 1 | pinustrb, tsugcana |
| AL | 2042 | Flatwoods/Blackland Prairie Margins | 33.21 | -88 | 3648 | 8 | juglcine, quercocc, querrubr |
| AL | 2122 | Gulf Barrier Islands and Coastal Marshes | 30.23 | -87.3 | 4788 | 4 | caryglab, lirituli, querfalc |
| AL | 2034 | Transition Hills | 34.83 | -88 | 4620 | 0 | pinuvirg |
| AL | 2089 | Southern Hilly Gulf Coastal Plain | 32.21 | -87.9 | 4195 | 7 | quercocc |
| AL | 2104 | Buhrstone/Lime Hills | 31.84 | -88 | 4331 | 4 | quervelu |
| AL | 2064 | Dissected Plateau | 34.13 | -87.3 | 3340 | -4 | tsugcana |
| AR | 2039 | Arkansas Valley Plains | 35.19 | -94.2 | 1462 | 9 | querpalu, querrubr, robipseu |
| AR | 2005 | Dissected Springfield Plateau-Elk River Hills | 36.22 | -93.9 | 7125 | 18 | tiliamer |
| AR | 2011 | Western Lowlands Holocene Meander Belts | 35.22 | -91.2 | 2748 | 6 | querpalu |
| AR | 2033 | Arkansas Valley Hills | 35.4 | -92.5 | 2244 | 20 | tiliamer |
| AR | 2036 | Arkansas River Floodplain | 35.25 | -93.6 | 1103 | 11 | querpalu, querrubr |
| AR | 2079 | Pleistocene Fluvial Terraces | 33.08 | -92.9 | 4179 | 3 | quervelu |
| AZ | 1906 | Grand Canyon | 36.26 | -112 | 82 | 0 | acernegu, pseumenz |
| AZ | 1876 | Arizona Strip Plateaus | 36.53 | -113 | 307 | 3 | pinupond |
| AZ | 1921 | Lower Grand Canyon | 35.94 | -113 | 93 | 0 | pinupond |
| AZ | 1953 | Lower Mogollon Transition | 34.23 | -112 | 172 | -1 | pinupond |
| AZ | 2060 | Lower Madrean Woodlands | 32.17 | -110 | 279 | -2 | pseumenz |
| BC | 970 | SBSmh | 53.1 | -122 | 270 | 13 | picemari |
| BC | 1050 | CDFmm | 49.02 | -124 | 217 | -4 | chamnoot, pinucont |
| BC | 978 | SBPSdc | 52.88 | -124 | 1079 | 26 | picemari |
| BC | 998 | IDFxm | 51.88 | -123 | 307 | 24 | piceglau |

| State | EcoID | Ecosystem Name | Lat | Long | Velocity | Habitat | Species with potentially valuable populations |
|---|---|---|---|---|---|---|---|
| BC | 1019 | IDFmw2 | 51.23 | -120 | 81 | 23 | piceglau |
| BC | 1053 | IDFxh2 | 50.53 | -121 | 121 | 11 | piceglau |
| BC | 1054 | IDFww1 | 50.64 | -122 | 42 | 17 | abieamab |
| BC | 1056 | IDFdk2 | 50.03 | -120 | 164 | 30 | piceglau |
| BC | 1060 | MSdm2 | 49.83 | -120 | 369 | 22 | larilyal |
| BC | 1075 | IDFdk5 | 50.71 | -116 | 82 | 13 | piceglau |
| BC | 1095 | IDFdm2 | 49.55 | -116 | 97 | 34 | popubals |
| CA | 1389 | Low Southern Cascades Mixed Conifer Forest | 41.62 | -122 | 227 | 10 | abieamab, pinucont, thujplic, tsughete |
| CA | 1459 | Klamath Juniper Woodland/Devils Garden | 41.96 | -121 | 626 | 3 | pinucont, poputric |
| CA | 1586 | Fort Bragg/Fort Ross Terraces | 39.09 | -124 | 1893 | 9 | abiegran, tsughete |
| CA | 1598 | Northeastern Sierra Mixed Conifer-Pine Forests | 39.54 | -120 | 337 | 1 | pinualbi, tsugmert |
| CA | 1679 | Central Sierra Lower Montane Forests | 38.34 | -120 | 234 | 7 | pinumont, poputric |
| CA | 1719 | Bay Terraces/Lower Santa Clara Valley | 37.51 | -122 | 561 | 5 | acernegu, pinupond |
| CA | 1808 | Interior Santa Lucia Range | 35.56 | -121 | 577 | 0 | pinupond, sequsemp |
| CA | 1446 | Western Klamath Low Elevation Forests | 41.32 | -124 | 119 | 0 | abiegran |
| CA | 1465 | Marble/Salmon Mountains-Trinity Alps | 41.27 | -123 | 126 | 2 | pinualbi |
| CA | 1470 | Outer North Coast Ranges | 40.03 | -123 | 321 | 12 | tsughete |
| CA | 1485 | Modoc Lava Flows and Buttes | 41.65 | -121 | 387 | 0 | pinucont |
| CA | 1495 | California Cascades Eastside Conifer Forest | 40.88 | -121 | 407 | -1 | pinucont |
| CA | 1555 | Foothill Ridges and Valleys | 39.54 | -122 | 248 | 3 | alnurubr |
| CA | 1570 | Coastal Franciscan Redwood Forest | 39.09 | -123 | 585 | -3 | abiegran |
| CA | 1605 | Northern Sierra Mid-Montane Forests | 39.64 | -121 | 233 | 2 | pinucont |
| CA | 1606 | Northern Sierra Upper Montane Forests | 39.17 | -120 | 280 | -4 | pinualbi |
| CA | 1646 | Sierra Valley | 39.72 | -120 | 236 | -8 | poputrem |
| CA | 1648 | Napa-Sonoma-Lake Volcanic Highlands | 38.56 | -123 | 684 | 3 | acernegu |
| CA | 1786 | Gabilan Range | 36.6 | -121 | 1020 | 1 | sequsemp |
| CA | 1843 | Eastern Sierra Mojavean Slopes | 35.52 | -118 | 112 | 4 | abieconc |
| CA | 1852 | South Valley Alluvium | 35.37 | -119 | 459 | 0 | pinulamb |
| CO | 1658 | Foothill Shrublands | 38.75 | -106 | 300 | 3 | abielasi, piceenge |
| CO | 1794 | Rolling Sand Plains | 38.41 | -103 | 2778 | 0 | junivirg, salinigr |
| CO | 1877 | San Luis Shrublands and Hills | 37.33 | -106 | 294 | -1 | abielasi |
| CO | 1699 | Moderate Relief Plains [1400-max m] | 39.88 | -104 | 1389 | -2 | fraxpenn |

| State | EcoID | Ecosystem Name | Lat | Long | Velocity | Habitat | Species with potentially valuable populations |
|---|---|---|---|---|---|---|---|
| CT | 1583 | Long Island Sound Coastal Lowland | 41.1 | -73 | 1363 | -14 | betupapy |
| DE | 1757 | Delmarva Uplands | 38.73 | -75.7 | 2783 | -12 | fraxnigr |
| DE | 1796 | Virginian Barrier Islands and Coastal Marshes | 37.72 | -75.5 | 4066 | -1 | popugran |
| FL | 2116 | Tallahasee Hills/Valdosta Limesink | 30.41 | -83.4 | 5007 | 23 | betunigr, caryglab, fagugran, lirituli, pinuechi, queralba |
| FL | 2120 | Gulf Coast Flatwoods | 30.06 | -85.1 | 5179 | 11 | acersacc, caryglab, lirituli, pinuechi, querfalc, querstel |
| FL | 2124 | Central Florida Ridges and Uplands | 28.82 | -81.9 | 4281 | -5 | betunigr, nysssylv, pinutaed, querfalc, querstel |
| FL | 2118 | Eastern Florida Flatwoods | 28.24 | -81.1 | 4796 | -5 | fraxamer, nysssylv, pinutaed, prunsero |
| FL | 2127 | Big Bend Coastal Marsh | 29.35 | -83.3 | 4897 | -2 | nysssylv |
| FL | 2137 | Southwestern Florida Flatwoods | 27.41 | -82 | 4625 | 0 | prunsero |
| GA | 2101 | Tifton Upland | 31.11 | -83.9 | 3983 | 25 | betunigr, pinuechi, queralba |
| GA | 1939 | Southern Outer Piedmont | 34.18 | -82.7 | 1733 | 4 | juglcine, pinuvirg |
| GA | 2015 | Southern Inner Piedmont | 33.9 | -84.6 | 829 | -3 | acerpens, pinurigi, pinustrb |
| GA | 2091 | Sea Island Flatwoods | 31.1 | -81.8 | 4560 | 19 | betunigr, caryglab, fraxamer, lirituli |
| GA | 2100 | Dougherty Plain | 31.21 | -85 | 3586 | 14 | juglnigr, junivirg, queralba |
| GA | 1926 | Southeastern Floodplains and Low Terraces | 33.05 | -84.2 | 3576 | 10 | juglcine, quercocc |
| GA | 2094 | Pine Mountain Ridges | 32.85 | -84.6 | 2405 | 3 | quercocc, querprin, querrubr |
| GA | 2250 | Southern Limestone/Dolomite Valleys and Low Rolling Hills [min-300 m] | 34.52 | -85.4 | 967 | -4 | acerspic, pinustrb |
| GA | 2083 | Coastal Plain Red Uplands | 32.57 | -83.4 | 2995 | 13 | quercocc, querprin |
| GA | 2102 | Bacon Terraces | 31.54 | -82.4 | 4467 | 15 | lirituli |
| GA | 2111 | Okefenokee Swamp | 30.73 | -82.3 | 5467 | 22 | caryglab |
| ID | 1460 | Foothill Shrublands-Grasslands | 43.53 | -115 | 270 | 0 | pinualbi |
| ID | 1499 | Eastern Snake River Basalt Plains | 43.38 | -113 | 749 | 0 | abielasi, piceenge, pinucont, poputric |
| ID | 1297 | Lower Clearwater Canyons | 46.43 | -116 | 337 | 3 | piceenge, pinucont |
| IL | 1732 | Chicago Lake Plain | 41.72 | -87.6 | 5111 | 2 | pinubank, popubals, prunpens, thujocci |
| IL | 1902 | Karstic Northern Ozarkian River Bluffs | 38.13 | -90 | 5822 | 21 | acersacr, fagugran, querbico |
| IL | 1717 | Valparaiso-Wheaton Morainal Complex | 41.78 | -87.9 | 5757 | 11 | pinubank |
| IL | 1720 | Rock River Hills | 42.08 | -89.7 | 5268 | 36 | pinubank |
| IL | 1842 | Wabash River Bluffs and Low Hills | 38.83 | -87.8 | 5112 | 24 | popugran |
| IL | 1850 | Southern Illinoian Till Plain | 38.59 | -89 | 5586 | 19 | popugran |
| IL | 1883 | Middle Mississippi Alluvial Plain | 38.17 | -89.9 | 5739 | 9 | fagugran, querbico |
| IN | 1840 | Wabash-Ohio Bottomlands | 38.04 | -88 | 4802 | 3 | popugran |
| IN | 1836 | Mitchell Plain | 38.35 | -86.2 | 5089 | 12 | fraxnigr |

| State | EcoID | Ecosystem Name | Lat | Long | Velocity | Habitat | Species with potentially valuable populations |
|---|---|---|---|---|---|---|---|
| KS | 1908 | Great Bend Sand Prairie | 37.97 | -98.7 | 5519 | -2 | fraxpenn, gledtria, junivirg, popudelt, quermacr |
| KS | 1790 | Rolling Plains and Breaks | 39.34 | -99.7 | 5265 | -1 | acersacc, ulmurubr |
| KS | 2236 | Flat to Rolling Plains [min-1100 m] | 38.68 | -101 | 4216 | 0 | acernegu, quermacr |
| KY | 1889 | Green River-Southern Wabash Lowlands | 37.77 | -87.4 | 4785 | 9 | fagugran, pinurigi, popugran |
| KY | 1946 | Western Pennyroyal Karst Plain | 36.79 | -86.9 | 4560 | 18 | pinurigi, popugran |
| KY | 1830 | Outer Bluegrass | 38.41 | -84.7 | 3975 | 15 | fraxnigr |
| KY | 1884 | Northern Forested Plateau Escarpment | 37.83 | -83.7 | 2648 | 13 | betualle |
| KY | 1930 | Caseyville Hills | 37.34 | -86.8 | 4795 | 16 | pinurigi, popugran |
| LA | 2076 | Floodplains and Low Terraces | 31.35 | -93.4 | 5023 | 17 | carycord, caryglab, caryovat, fagugran, juglnigr, lirituli, quermacr |
| LA | 2129 | Baton Rouge Terrace | 30.49 | -90.9 | 5821 | 14 | betunigr, fagugran, lirituli, queralba |
| LA | 2135 | Texas-Louisiana Coastal Marshes | 29.82 | -93.3 | 5987 | 7 | fraxamer, pinuechi, querfalc, querstel |
| LA | 2131 | Inland Swamps | 30.07 | -91.2 | 5726 | 7 | acersacc, fraxamer |
| LA | 2066 | Tertiary Uplands | 32.68 | -93.9 | 4477 | 13 | quervelu |
| LA | 2090 | Red River Bottomlands | 32.39 | -93.5 | 4336 | 5 | quervelu |
| LA | 2113 | Southern Backswamps | 30.99 | -91.7 | 6659 | 9 | acersacc |
| LA | 2114 | Southern Holocene Meander Belts | 30.53 | -91.3 | 5930 | 7 | acersacc |
| MB | 745 | Shilo | 49.89 | -99.4 | 5222 | 4 | picemari, pinubank |
| MB | 756 | Carberry | 49.95 | -99.4 | 5692 | 5 | picemari |
| MD | 1722 | Piedmont Uplands | 39.28 | -77 | 1187 | 7 | fraxnigr |
| ME | 1354 | Midcoast | 44.08 | -69.5 | 1670 | 2 | picemari |
| ME | 1412 | Gulf of Maine Coastal Lowland | 43.18 | -70.7 | 1038 | 2 | abiebals |
| MI | 1580 | Saginaw Lake Plain | 43.51 | -83.5 | 3638 | -12 | abiebals, pinubank, thujocci |
| MI | 1635 | Maumee Lake Plain | 41.77 | -83.7 | 4991 | -17 | betualle, fraxnigr, larilari, piceglau, popubals, thujocci |
| MI | 1572 | Cadillac Hummocky Moraines | 43.85 | -85.3 | 6130 | -10 | picemari |
| MI | 1601 | Lansing Loamy Plain | 42.94 | -84.6 | 3374 | -9 | betualle, fraxnigr, pinubank, thujocci |
| MI | 1625 | Interlobate Dead Ice Moraines | 42.45 | -84.2 | 3995 | -6 | piceglau, thujocci |
| MI | 1571 | Newaygo Barrens | 43.86 | -85.9 | 5624 | -39 | abiebals, acerspic |
| MI | 1688 | Oak Openings | 41.76 | -83.7 | 5088 | -17 | betualle, fraxnigr, larilari, popubals |
| MI | 1483 | Onaway Moraines | 45.14 | -84.1 | 3226 | 0 | sorbamer |
| MI | 1640 | Lake Michigan Moraines | 42.64 | -86 | 3652 | -11 | pinubank |
| MI | 1677 | Battle Creek/Elkhart Outwash Plain | 41.87 | -85.7 | 4430 | -1 | pinubank, thujocci |
| MI | 1547 | Tawas Lake Plain | 44.13 | -84 | 5044 | -7 | abiebals, picemari |

| State | EcoID | Ecosystem Name | Lat | Long | Velocity | Habitat | Species with potentially valuable populations |
|---|---|---|---|---|---|---|---|
| MI | 1509 | Manistee-Leelanau Shore | 44.9 | -85.6 | 3473 | -20 | piceglau |
| MI | 1553 | Platte River Outwash | 44.59 | -85.9 | 4305 | -34 | picemari |
| MN | 1316 | Beach Ridges and Sand Deltas | 47.64 | -96.8 | 5963 | 8 | abiebals |
| MO | 1968 | Black River Hills Border | 36.96 | -90.5 | 7323 | 31 | acersacr, caryglab, juglcine, quercocc, tiliamer |
| MO | 1990 | White River Hills | 36.63 | -92.8 | 8368 | 22 | acersacr, juglcine, querbico |
| MO | 1898 | Eastern Ozark Border | 37.9 | -90.3 | 6975 | 33 | querbico |
| MO | 1770 | River Hills | 39.12 | -91.1 | 5882 | 44 | fraxnigr |
| MS | 1984 | Bluff Hills | 34.22 | -90.5 | 5048 | 9 | acersacr, juglcine, lirituli, querpalu, querprin, querrubr, tiliamer |
| MS | 1972 | Loess Plains | 34.8 | -89.6 | 5462 | 6 | acersacr |
| MS | 2109 | Southern Rolling Plains | 31.45 | -90.8 | 6589 | 3 | acersacc, caryglab, junivirg, quervelu |
| MS | 2043 | Blackland Prairie | 33.28 | -88.7 | 3394 | 8 | juglcine, quercocc, querrubr |
| MS | 2021 | Northern Pleistocene Valley Trains | 34.19 | -90.4 | 4758 | 7 | lirituli, querpalu |
| MS | 2037 | Northern Backswamps | 33.13 | -90.9 | 5596 | 0 | quervelu |
| MT | 1277 | Camas Valley | 47.63 | -115 | 214 | 5 | betupapy, thujplic |
| MT | 1231 | Salish Mountains | 48.3 | -115 | 352 | 16 | larilyal |
| MT | 1303 | Rattlesnake-Blackfoot-South Swan-Northern Garnet-Sapphire Mountains | 46.82 | -113 | 461 | 4 | larilyal |
| NC | 1871 | Southern Shale Valleys | 35.19 | -84.3 | 665 | 4 | acerpens, acerspic, betulent, pinurigi, pinustrb, querbico, tsugcana |
| NC | 1973 | Eastern Blue Ridge Foothills | 35.77 | -81.6 | 338 | 12 | acerpens |
| NC | 1817 | Rolling Coastal Plain | 36.54 | -77.6 | 3347 | -2 | popugran |
| NC | 1859 | Triassic Basins | 36 | -79.2 | 2224 | 6 | betulent, pinurigi |
| NC | 2003 | Broad Basins | 35.25 | -83.3 | 276 | 3 | betualle |
| NC | 1905 | Southern Crystalline Ridges and Mountains [850-max m] | 35.63 | -82.6 | 589 | 4 | betupapy |
| NC | 1914 | Carolinian Barrier Islands and Coastal Marshes | 34.94 | -76.8 | 4221 | 1 | querrubr |
| ND | 1304 | Glacial Lake Basins | 46.8 | -99 | 5354 | 2 | popubals, prunpens, tiliamer |
| ND | 1325 | Drift Plains | 47.1 | -98.7 | 5972 | 2 | tiliamer |
| ND | 1310 | Glacial Lake Deltas | 47.79 | -99.8 | 4697 | 2 | prunpens |
| ND | 1315 | Miissouri Coteau Slope | 47.83 | -102 | 5831 | 1 | prunpens |
| ND | 1332 | River Breaks | 45.97 | -102 | 3876 | -1 | prunpens |
| ND | 1348 | Missouri Plateau | 46.53 | -103 | 4748 | -1 | prunpens |

| State | EcoID | Ecosystem Name | Lat | Long | Velocity | Habitat | Species with potentially valuable populations |
|---|---|---|---|---|---|---|---|
| ND | 1349 | Missouri Coteau | 46.46 | -99.8 | 6381 | 0 | popubals |
| NE | 1686 | Niobrara River Breaks | 42.79 | -99.8 | 5679 | 0 | ostrvirg |
| NE | 1700 | Holt Tablelands | 42.54 | -98.5 | 5358 | 0 | ostrvirg |
| NH | 1428 | Gulf of Maine Coastal Plain | 42.87 | -71.4 | 1243 | 4 | abiebals |
| NJ | 1641 | Glaciated Triassic Lowlands | 40.88 | -74.2 | 926 | -19 | betupapy, prunpens |
| NJ | 1662 | Passaic Basin Freshwater Wetlands | 40.76 | -74.4 | 1440 | -19 | prunpens |
| NJ | 1663 | Hackensack Meadowlands | 40.72 | -74.1 | 705 | -7 | betupopu |
| NJ | 1689 | Inner Coastal Plain | 40 | -74.8 | 1573 | -7 | betupopu |
| NJ | 1696 | Pine Barrens | 39.75 | -74.6 | 2268 | -8 | betupopu |
| NJ | 1615 | Barrier Islands/Coastal Marshes | 39.93 | -74 | 2344 | -4 | betupopu |
| NM | 1865 | Pinyon-Juniper Woodlands and Savannas | 35.46 | -105 | 665 | -1 | acernegu, popudelt |
| NM | 1957 | Mesa de Maya/Black Mesa | 36.97 | -103 | 1132 | 1 | acernegu, popudelt |
| NM | 1983 | Madrean Lower Montane Woodlands [1800-max m] | 33.25 | -108 | 348 | 3 | acernegu |
| NM | 2024 | Lava Malpais | 34.11 | -107 | 667 | 1 | pinupond |
| NM | 2028 | Central New Mexico Plains [1850-max m] | 34.62 | -106 | 733 | -1 | acernegu |
| NV | 1607 | Sierra Nevada-Influenced Semiarid Hills and Basins | 39.27 | -120 | 146 | -2 | poputrem, poputric |
| NV | 1564 | Upper Lahontan Basin | 40.87 | -118 | 300 | 0 | poputrem |
| NV | 1642 | Sierra Nevada-Influenced Ranges | 38.38 | -119 | 165 | -3 | pinumont |
| NV | 1759 | Tonopah Uplands | 37.55 | -117 | 541 | -8 | poputric |
| NY | 1498 | Hudson Valley | 42.52 | -73.8 | 602 | -5 | abiebals, picerube |
| NY | 1479 | Ontario Lowlands | 43.19 | -77 | 1778 | -47 | betupopu, piceglau, picerube |
| NY | 1521 | Mohawk Valley | 42.99 | -74.8 | 905 | -2 | piceglau |
| OH | 1684 | Clayey High Lime Till Plains | 40.89 | -84.3 | 6131 | -7 | popubals, thujocci |
| OH | 1805 | Ohio/Kentucky Carboniferous Plateau | 38.64 | -82.8 | 3189 | 11 | betulent, pinustrb, poputrem, tsugcana |
| OH | 1812 | Knobs-Lower Scioto Dissected Plateau | 38.8 | -83.2 | 3834 | 12 | poputrem |
| OH | 1741 | Paulding Plains | 41.26 | -84.4 | 5802 | 3 | fraxnigr, larilari |
| OH | 1630 | Low Lime Drift Plain | 41.21 | -81 | 4499 | -6 | abiebals |
| OK | 1992 | Rolling Red Hills | 35.92 | -99.3 | 5528 | 0 | junivirg, quermueh |
| OK | 2000 | Salt Plains | 36.78 | -98.2 | 5213 | -3 | popudelt |
| OK | 2249 | Osage Cuestas [min-300 m] | 36.99 | -95.5 | 5037 | 17 | querbico, tiliamer |
| OK | 1947 | Northern Cross Timbers | 35.75 | -96.5 | 4870 | -5 | carycord |

| State | EcoID | Ecosystem Name | Lat | Long | Velocity | Habitat | Species with potentially valuable populations |
|---|---|---|---|---|---|---|---|
| OK | 1996 | Pleistocene Sand Dunes | 36.21 | -98.8 | 5314 | -2 | quermueh |
| OK | 2044 | Lower Canadian Hills | 34.88 | -96 | 3621 | 18 | querpalu |
| ON | 830 | Southern Horseshoe Moraine | 43.04 | -81.1 | 5704 | -59 | picemari |
| ON | 808 | Smith Falls Plain | 44.9 | -75.9 | 2175 | -38 | picerube |
| OR | 1281 | Oak/Conifer Foothills | 45.67 | -121 | 229 | -3 | abieamab, abielasi, lariocci, piceenge, pinualbi, pinucont, tsughete, tsugmert |
| OR | 1313 | Ponderosa Pine/Bitterbrush Woodland | 44.49 | -122 | 184 | 5 | lariocci, piceenge, thujplic, tsughete, tsugmert |
| OR | 1361 | Continental Zone Foothills | 44.36 | -118 | 354 | 1 | abiegran, lariocci |
| OR | 1373 | Pumice Plateau | 43.12 | -121 | 534 | 3 | tsugmert |
| OR | 1202 | Coastal Lowlands | 44.02 | -124 | 761 | -7 | abieamab, chamnoot |
| OR | 1318 | Umatilla Dissected Uplands | 45.37 | -119 | 344 | 0 | betupapy, lariocci |
| OR | 1353 | Cold Basins | 44.45 | -119 | 728 | 16 | lariocci |
| OR | 1422 | Oak Savanna Foothills | 42.39 | -123 | 207 | 3 | abiegran, thujplic |
| OR | 1438 | Klamath/Goose Lake Basins | 42.14 | -121 | 357 | 2 | pinucont, pinumont |
| OR | 1209 | Volcanics | 46.03 | -123 | 481 | 4 | abielasi |
| OR | 1268 | Valley Foothills | 44.71 | -123 | 418 | -6 | abieamab |
| OR | 1279 | Mid-Coastal Sedimentary | 43.8 | -124 | 955 | -2 | abieamab |
| OR | 1327 | Canyons and Dissected Uplands | 45.71 | -117 | 174 | 2 | abielasi |
| OR | 1344 | Blue Mountain Basins | 45.35 | -118 | 230 | 4 | betupapy |
| OR | 1425 | Rogue/Illinois/Scott Valleys | 42.12 | -123 | 190 | -3 | poputrem |
| OR | 1429 | High Desert Wetlands | 42.93 | -119 | 405 | 0 | poputrem |
| OR | 1447 | Border High-Siskiyous | 42.01 | -123 | 278 | 4 | piceenge |
| OR | 1454 | Fremont Pine/Fir Forest | 42.27 | -121 | 502 | 5 | pinualbi |
| PA | 1673 | Northern Shale Valleys | 40 | -77.6 | 720 | -4 | betupapy, betupopu, larilari, picerube, pinuresi, sorbamer |
| PA | 1674 | Triassic Lowlands | 39.92 | -76.1 | 1147 | -8 | betupopu, poputrem, prunpens |
| PA | 1556 | Erie/Ontario Lake Plain | 42.57 | -79.5 | 1929 | -21 | betupopu, thujocci |
| PA | 1633 | Northern Sandstone Ridges | 39.79 | -78.1 | 1068 | -5 | larilari |
| PA | 1645 | Trap Rock and Conglomerate Uplands | 40.05 | -76 | 1202 | -12 | larilari, prunpens |
| PA | 1707 | Pittsburgh Low Plateau | 40.78 | -79.7 | 2579 | -9 | popubals |
| RI | 1528 | Cape Cod/Long Island | 41.3 | -71.6 | 2327 | -5 | betupapy |
| SC | 2051 | Sea Islands/Coastal Marsh | 32.1 | -80.7 | 4242 | 22 | fraxamer, pinuechi |
| SC | 2233 | Sand Hills [min-300 m] | 33.77 | -81.7 | 3070 | 10 | querprin |

| State | EcoID | Ecosystem Name | Lat | Long | Velocity | Habitat | Species with potentially valuable populations |
|---|---|---|---|---|---|---|---|
| SC | 1977 | Carolina Flatwoods | 33.8 | -79.3 | 4186 | -5 | quercocc |
| SC | 2007 | Atlantic Southern Loam Plains | 33.07 | -81.5 | 3591 | 12 | quercocc |
| SD | 1558 | James River Lowland | 43.91 | -97.9 | 4930 | 0 | tiliamer |
| SD | 1649 | Southern River Breaks | 43.03 | -98.8 | 4587 | 0 | ostrvirg |
| SD | 1654 | Ponca Plains | 43.17 | -99.3 | 4664 | 0 | ostrvirg |
| SK | 528 | Sturgeon River Plain | 53.61 | -107 | 3742 | 43 | abiebals |
| SK | 539 | Shellbrook Plain | 53.28 | -106 | 3474 | 18 | picemari, pinubank |
| SK | 549 | Nisbet Plain | 52.97 | -106 | 2884 | 9 | larilari, pinubank |
| SK | 557 | Prince Albert Plain | 53.04 | -105 | 3394 | 9 | larilari |
| SK | 645 | Beechy Hills | 50.92 | -107 | 4254 | -1 | populbals |
| SK | 652 | Eyebrow Plain | 50.86 | -106 | 4486 | -1 | populbals |
| TN | 1878 | Southern Limestone/Dolomite Valleys and Low Rolling Hills [300-max m] | 36.44 | -82.8 | 497 | 2 | acerpens, betualle, betulent |
| TN | 1870 | Southern Sandstone Ridges | 35.74 | -83.4 | 1005 | 10 | acerpens, betualle, sorbamer |
| TN | 1932 | Plateau Escarpment | 35.86 | -85.3 | 1844 | 8 | acerpens |
| TN | 1891 | Southern Dissected Ridges and Knobs | 36.14 | -83.2 | 486 | 8 | sorbamer |
| TN | 1936 | Eastern Highland Rim | 36.07 | -85.9 | 2490 | 3 | pinurigi, querbico |
| TN | 2008 | Northern Hilly Gulf Coastal Plain | 35.12 | -88.9 | 5691 | 10 | pinuvirg |
| TN | 1965 | Outer Nashville Basin | 35.82 | -86.5 | 2927 | -1 | acersacr, querbico |
| TN | 1970 | Western Highland Rim | 35.91 | -87.6 | 4899 | 2 | acersacr, querbico |
| TN | 1937 | Cumberland Plateau | 36.01 | -85 | 1580 | 7 | betualle |
| TN | 2006 | Inner Nashville Basin | 35.83 | -86.5 | 2605 | -2 | acersacr |
| TX | 2048 | Grand Prairie | 33.52 | -95.4 | 4165 | -5 | acersacc, carpcaro, caryovat, nyssaqua, nysssylv, quermich, taxodist |
| TX | 1959 | Canadian/Cimarron Breaks | 36.36 | -100 | 4797 | -1 | juglnigr, junivirg, populdelt, salinigr |
| TX | 2125 | Flatwoods | 30.46 | -94 | 7961 | 6 | caryglab, fagugran, juglnigr, lirituli |
| TX | 2110 | Southern Post Oak Savanna | 29.98 | -97.1 | 4327 | 0 | gledtria, juglnigr, nysssylv |
| TX | 2126 | Balcones Canyonlands | 29.95 | -98.9 | 6146 | -7 | juglnigr, platocci, prunsero |
| TX | 2088 | Northern Post Oak Savanna | 33 | -95.7 | 4542 | 4 | caryglab, nysssylv |
| TX | 2123 | San Antonio Prairie | 30.64 | -96.6 | 7357 | 0 | gledtria, juglnigr |
| TX | 2041 | Caprock Canyons, Badlands, and Breaks | 33.62 | -101 | 3240 | -1 | salinigr |
| TX | 2085 | Eastern Cross Timbers | 33.35 | -97 | 5192 | -7 | quervelu |
| TX | 2106 | Limestone Cut Plain | 31.55 | -98 | 7265 | -6 | juglnigr |

| State | EcoID | Ecosystem Name | Lat | Long | Velocity | Habitat | Species with potentially valuable populations |
|---|---|---|---|---|---|---|---|
| TX | 2119 | Edwards Plateau Woodland | 30.52 | -99.6 | 6072 | 1 | juglnigr |
| TX | 2128 | Northern Humid Gulf Coastal Prairies | 29.63 | -95.1 | 5516 | -2 | quervelu |
| TX | 2142 | Southern Subhumid Gulf Coastal Prairies | 27.93 | -97.6 | 4641 | 4 | fraxpenn |
| UT | 1413 | Semiarid Foothills | 40.57 | -113 | 243 | -5 | piceenge |
| UT | 1775 | Escarpments | 38.88 | -110 | 357 | -9 | abielasi, piceenge |
| UT | 1682 | Mountain Valleys | 39.22 | -112 | 236 | -4 | abielasi |
| VA | 1807 | Northern Inner Piedmont | 36.89 | -79.6 | 778 | 4 | acerpens, acerspic, betulent, pinustrb |
| VA | 1755 | Northern Igneous Ridges | 38.47 | -78.5 | 614 | 4 | acerspic, sorbamer |
| VA | 1756 | Northern Sedimentary and Metasedimentary Ridges | 38.75 | -78.4 | 855 | 4 | sorbamer |
| VA | 1776 | Chesapeake-Pamlico Lowlands and Tidal Marshes | 36.85 | -76.3 | 3761 | -4 | popugran |
| VA | 1826 | Northern Outer Piedmont | 36.82 | -78.1 | 2591 | -4 | pinurigi, popugran |
| WA | 1211 | Okanogan Pine/Fir Hills | 48.46 | -120 | 127 | 15 | abieamab, chamnoot |
| WA | 1267 | Palouse Hills | 47 | -117 | 688 | 9 | betupapy, lariocci, piceenge |
| WA | 1201 | North Cascades Lowland Forests | 48.38 | -122 | 77 | 5 | pinualbi |
| WA | 1212 | Okanogan Valley | 48.38 | -120 | 151 | 3 | abiegran, thujplic |
| WA | 1199 | Low Olympics | 47.88 | -124 | 189 | 1 | abielasi |
| WA | 1206 | Eastern Puget Riverine Lowlands | 47.94 | -122 | 270 | -7 | abieamab |
| WA | 1207 | Olympic Rainshadow | 48.05 | -123 | 257 | -1 | abielasi |
| WA | 1248 | Yakima Plateau and Slopes | 46.46 | -121 | 218 | 2 | chamnoot |
| WA | 1250 | Spokane Valley Outwash Plains | 47.85 | -117 | 287 | 11 | tsughete |
| WA | 1285 | Dissected Loess Uplands | 46.45 | -117 | 404 | -3 | lariocci |
| WA | 1295 | Deep Loess Foothills | 46.14 | -118 | 232 | 1 | betupapy |
| WV | 1678 | Northern Limestone/Dolomite Valleys | 39.55 | -78 | 619 | 7 | acerpens, acerspic, betualle, betupopu, fraxnigr, picerube, pinuresi, sorbamer |
| WV | 1765 | Monongahela Transition Zone | 39.24 | -81.2 | 1717 | 12 | acerspic, poputrem |
| WV | 1841 | Greenbriar Karst | 37.7 | -80.7 | 489 | 8 | picerube |
| WY | 1539 | Black Hills Foothills | 44.28 | -104 | 648 | 1 | ostrvirg |
| WY | 1593 | Sub-Irrigated High Valleys | 42.21 | -110 | 558 | -1 | pinualbi |
